# Computational modeling of trans-synaptic nanocolumns, a modulator of synaptic transmission

**DOI:** 10.3389/fncom.2022.969119

**Published:** 2022-09-28

**Authors:** Xiaoting Li, Gabriel Hémond, Antoine G. Godin, Nicolas Doyon

**Affiliations:** ^1^Department of Mathematics and Statistics, Université Laval, Québec City, QC, Canada; ^2^Department of Psychiatry and Neuroscience, Université Laval, Québec City, QC, Canada; ^3^CERVO Brain Research Centre, Québec City, QC, Canada; ^4^Department of Physics, Université Laval, Québec City, QC, Canada

**Keywords:** receptor kinetics, glutamate, tetrapartite synapse, synaptic current, trans-synaptic nanocolumn, Monte Carlo simulation, synaptic organization, neurotransmitter molecule diffusion

## Abstract

Understanding synaptic transmission is of crucial importance in neuroscience. The spatial organization of receptors, vesicle release properties and neurotransmitter molecule diffusion can strongly influence features of synaptic currents. Newly discovered structures coined trans-synaptic nanocolumns were shown to align presynaptic vesicles release sites and postsynaptic receptors. However, how these structures, spanning a few tens of nanometers, shape synaptic signaling remains little understood. Given the difficulty to probe submicroscopic structures experimentally, computer modeling is a useful approach to investigate the possible functional impacts and role of nanocolumns. In our *in silico* model, as has been experimentally observed, a nanocolumn is characterized by a tight distribution of postsynaptic receptors aligned with the presynaptic vesicle release site and by the presence of trans-synaptic molecules which can modulate neurotransmitter molecule diffusion. In this work, we found that nanocolumns can play an important role in reinforcing synaptic current mostly when the presynaptic vesicle contains a small number of neurotransmitter molecules. Our work proposes a new methodology to investigate *in silico* how the existence of trans-synaptic nanocolumns, the nanometric organization of the synapse and the lateral diffusion of receptors shape the features of the synaptic current such as its amplitude and kinetics.

## 1. Introduction

Understanding the determinants of synaptic transmission is important in the field of neuroscience because it is essential for understanding normal brain function and for studying neurological disorders and diseases. Although many features of synaptic transmission, such as the number of neurotransmitter molecules and receptor properties (Sayer et al., 1990; Karunanithi et al., 2002; Kilman et al., 2002), have been extensively investigated, we are yet to obtain a complete picture. To fully investigate synaptic transmission, one needs to simultaneously consider all the components of the tetrapartite synapse (Chelini et al., 2018) including the presynaptic vesicle, the postsynaptic receptors, the geometry of the extracellular space (Syková and Nicholson, 2008; Godin et al., 2017) which shapes the diffusion of neurotransmitter molecules, and the astrocytes which control the chemical environment of the synapse (Chung et al., 2015).

Nanocolumns are submicroscopic structures which span the presynaptic, synaptic, and postsynaptic spaces (Tang et al., 2016; Chen et al., 2018; Sarkar et al., 2022). These structures were shown to align the postsynaptic receptors to the presynaptic vesicle docking sites and can be characterized by the presence of molecules spanning the synaptic cleft (Zuber et al., 2005; Sigrist and Petzoldt, 2016; Tang et al., 2016). Efforts have been made to understand the mechanisms of nanocolumn formation and persistence. Receptors diffuse in the postsynaptic dense area (PSD) (MacGillavry et al., 2011; Biederer et al., 2017) and can be fixed at specific locations by anchoring proteins such as PSD-95 (Chen et al., 2000; Choquet and Triller, 2003; Keith and El-Husseini, 2008; Yoo et al., 2019) or gephyrin (Savtchenko et al., 2000; Chen et al., 2008). The impact of trans-synaptic molecules on receptor anchoring was hypothesized to explain nanocolumn formation (Tang et al., 2016; Yang et al., 2021). An alternative mechanism would be that receptors located under the sites of vesicles are more likely to open and that current through a receptor would favor anchoring (Regalado et al., 2006; Glasgow et al., 2018).

Although the question of how trans-synaptic nanocolumns arise is interesting and important (Chen et al., 2018), in the present paper we focus instead on the potential functional impact of these structures and specifically on how they shape synaptic currents. We develop a new *in silico* model to study the influence of parameters related to nanocolumn properties on the amplitude, rise time, and decay time of synaptic currents. We model an excitatory synapse with glutamate neurotransmitter molecules and AMPA receptors. However, our approach could be directly transposed to any neurotransmitter molecule and receptor pair, as long as the state transition kinetics of the receptors are well-characterized and the diffusion coefficient of the transmitter is known. A computational model describing the role of nanocolumns has been developed in Ventriglia (2011). They described each trans-synaptic filament individually by a cylinder. The net effect of these cylinders was to slow diffusion of neurotransmitter molecules in the plane parallel to the synapse. In the present work, we take the alternative approach of homogenizing the effect of these trans-synaptic filaments. We describe their impact by a decrease of the diffusion coefficient in the *xy* plane where these filaments are present. By avoiding a complex geometrical description, this new approach speeds up simulations allowing the investigation of many parameters.

**Figure 1:**
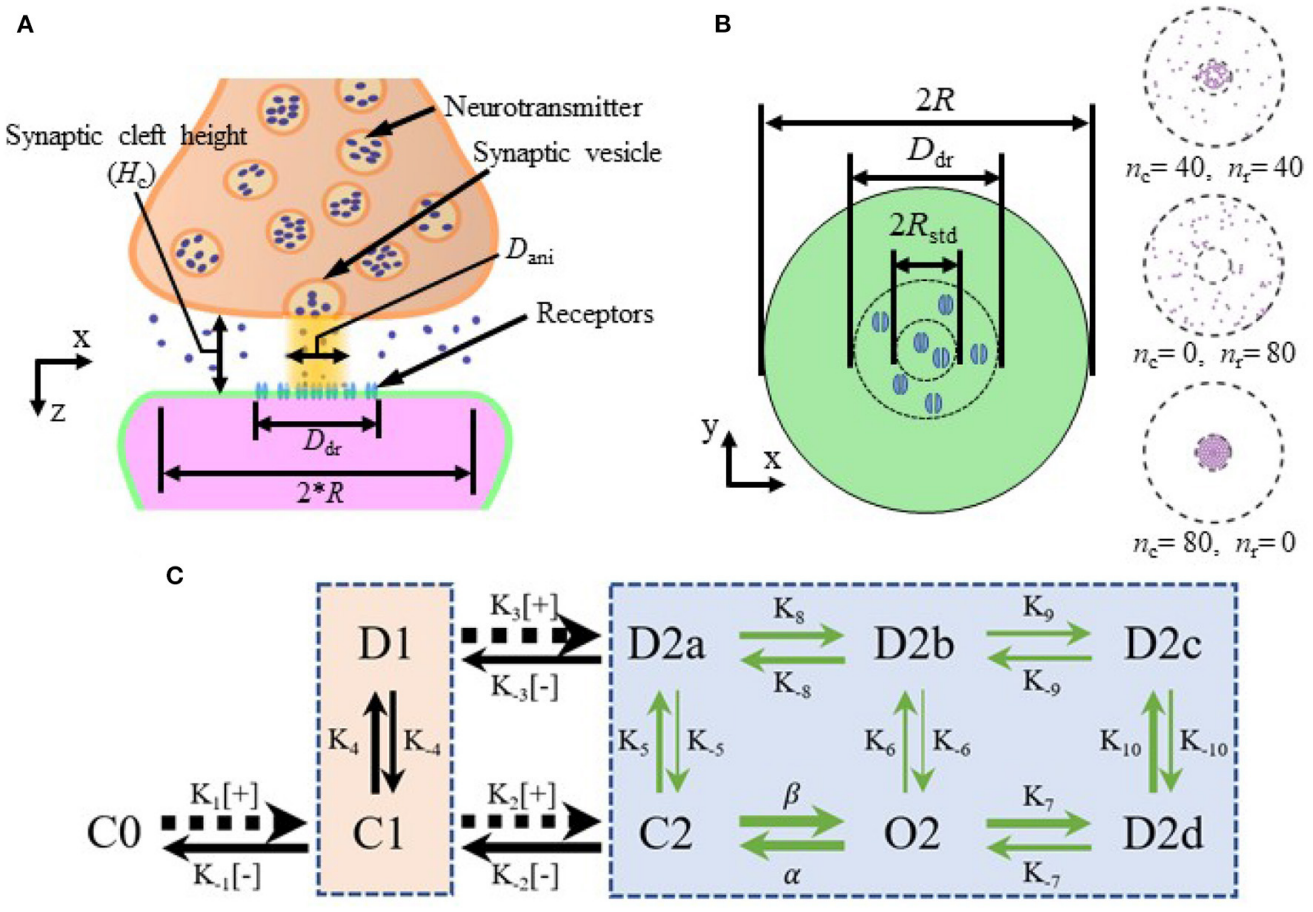
Schematic of the synapse and kinetics of AMPA receptors. **(A)** Schematic of the synapse. **(B)** Top view of the postsynaptic side of the synaptic cleft with height *H*_c_ and examples of receptor distributions (right side) with different numbers of receptors inside and outside of the nanocolumn. Here, *R* is the radius of the synaptic space, *D*_dr_ is the diameter of postsynaptic density (PSD), *D*_ani_ is the diameter of the zone where trans-synaptic filaments are present, *R*_std_ is the standard deviation of distribution of receptors located inside the nanocolumn, *n*_c_ is the number of receptors inside the nanocolumn and *n*_r_ is the number of receptors randomly distributed outside the nanocolumn. **(C)** Kinetics of AMPA receptors. C, closed; O, open; D, desensitized; [+], capturing a glutamate molecule; [−], releasing one. The number represents the number of bound glutamate molecule(s) in a given state. The thickness of the arrow is larger when the transition rate is larger and the values of the transition rates are as follows (in units of *M*^−1^*s*^−1^ for *K*_1_, *K*_2_, *K*_3_, and of *s*^−1^ for the rest): $K_{1}=1.8412\times 10^{7}$, $K_{-1}=4.323\times 10^{3}$, $K_{2}=4.000\times 10^{6}$, $K_{-2}=1.7201\times 10^{4}$, $K_{3}=1.9863\times 10^{7}$, $K_{-3}=1.168\times 10^{3}$, β = 5.1690 × 10^4^, α = 1.0082 × 10^4^, *K*_4_ = 885.990, *K*_−4_ = 280.350, *K*_5_ = 449.033, *K*_−5_ = 1.944, *K*_6_ = 2.797, $K_{-6}=3.9497\times 10^{-2}$, $K_{7}=1.380\times 10^{3}$, *K*_−7_ = 421.849, *K*_8_ = 848.141, *K*_−8_ = 538.920, *K*_9_ = 51.700, *K*_−9_ = 29.164, *K*_10_ = 939.000, *K*_−10_ = 24.463 (Budisantoso et al., 2013). The beige and blue boxes separate the states with one and two bound glutamate molecules, respectively.

Nanocolumns place postsynaptic receptors where the concentration of neurotransmitter molecules is greatest. We thus hypothesized that the presence of the nanocolumns could increase the maximal proportion of open receptors and thus increase peak synaptic current (Scimemi and Beato, 2009). Given the proximity of receptors to vesicle docking sites, the presence of nanocolumns could also decrease the delay between the vesicle opening and the binding of neurotransmitter molecules to postsynaptic channels (Tang et al., 2016) which could in principle decrease the rise time of synaptic currents. Our results confirm that the placement of postsynaptic receptors has an important effect on the rise time. Such an effect has also been investigated in Ventriglia (2011). Besides impacting the placement of postsynaptic receptors, nanocolumns are also linked to the presence of trans-synaptic molecules crowding the extracellular space. These molecules could hinder the diffusion of neurotransmitter molecules and possibly favor their binding to receptors located directly under the vesicle, potentially further increasing peak synaptic current. Figure 1A shows a schematic of the organization of the synapse and the relative position of its different morphological components.

Due to the small spatial and temporal scales, the nanocolumns and the diffusion of the neurotransmitter molecule are very difficult to investigate experimentally (Zheng et al., 2017). *In silico* simulations are thus ideal to study the relationships between parameters related to nanocolumns and features that shape synaptic current. Several computational methods have been developed to simulate the diffusion of neurotransmitter molecules in the synaptic cleft, their binding to receptors and the resulting synaptic current (Clements, 1996; Franks et al., 2002; Savtchenko and Rusakov, 2007). For example, Savtchenko and Rusakov (2007) performed simulations in which they investigated the impact of synaptic cleft height arguing that physiological values tend to maximize synaptic glutamatergic current.

Many of the previous modeling works relied on several assumptions. A common approach is to model neurotransmitter molecule diffusion with a continuous concentration function. Such a function depends on both space and time and can be computed by solving the heat equation (Savtchenko and Rusakov, 2007; Montes et al., 2015). The advantage of this approach is that it is relatively computationally inexpensive to calculate the temporal evolution of neurotransmitter molecule concentration. This approximation however implicitly assumes that the capture and release of neurotransmitter molecules by the receptors has no impact on the time course of neurotransmitter molecule concentration. This is a reasonable approximation when the number of neurotransmitter molecules is very large compared to the number of receptors but leads to inaccurate predictions otherwise. A second shortcoming of this approach is that it neglects the stochastic nature of neurotransmitter molecule diffusion and thus may fail to capture some of the variability of the system. Another common simplification of previous mathematical models is the assumption of axial symmetry with respect to either the distribution of neurotransmitter molecules, the distribution of postsynaptic receptors, or of the electric field. This simplification may fail to capture the impact of the random receptor distribution.

Here, we use a Monte Carlo approach (Franks et al., 2002; Montes et al., 2015) to track the position of each neurotransmitter molecule and the state of each individual receptor. We thus avoid the above mentioned simplifications. In this work, we describe an excitatory synapse with glutamate molecules as neurotransmitters and AMPA receptors. We believe that the modeling formalism developed in this work could be transposed to other synaptic types or channels. When describing AMPA postsynaptic receptors, we consider two binding sites and nine possible different states, including three closed states, one open state and five desensitized states as in Budisantoso et al. (2013) and Kleinle et al. (1996) (Figure 1C). As glutamate molecules are negatively charged, their displacements in the synaptic cleft can be influenced by the electric field resulting from synaptic currents (Savtchenko et al., 2004). It is also known that the electric field within the synapse can decrease the synaptic driving force leading to a decoupling between the current and the conductance. Since the electric field within the cleft can influence glutamate diffusion and trans-synaptic currents, we described it without assuming its axial symmetry.

We investigate the influence of several parameters on synaptic currents including: the height of the synaptic cleft, the number of receptors, the number of released glutamate neurotransmitter molecules, and other parameters related to the nanocolumn itself (see Table 1). In our model, these parameter include the radius of the nanocolumn as well as the number receptors inside it. The presence of nanocolumns can also be accompanied by filament like trans-synaptic molecules (Ventriglia, 2011; Tang et al., 2016) which hinder the diffusion of neurotransmitter molecules in the plane parallel to the postsynaptic membrane. Our model accounted for this possibility by including a parameters reducing the diffusion coefficient in the *xy* plane where filaments are present (see Figure 1A). Avoiding the geometrical description of individual filaments decreases the simulation time and allows to test several hypotheses. Given the cylindrical geometry of the filaments, they make the diffusion anisotropic, hindering it in the *xy* plan but not affecting it in the *z* axis. We thus describe the effect of the filament by an anisotropy coefficient.

**Table 1:** Values of parameters and constants.

**Symbol**	**Description**	**Unit**	**Value**	**References**
**Parameters and constants**				
*R*	Radius of the synaptic space	nm	1,000	Savtchenko and Rusakov, 2007
*H*_c_	Synaptic cleft height	nm	5–50	Kleinle et al., 1996
*r*_ves_	Radius of the vesicle	nm	20	Kleinle et al., 1996
nnt	Number of glutamate neurotransmitter molecules	No unit	200–20,000	Budisantoso et al., 2013
ani	Anisotropy coefficient	No unit	0–0.9	
*n*_r_	Number of receptors outside the nanocolumn	No unit	0–80	Savtchenko and Rusakov, 2007
*n*_c_	Number of receptors inside the nanocolumn	No unit	0–80	Savtchenko and Rusakov, 2007
*D*_dr_	Diameter of postsynaptic density (PSD)	nm	400	Savtchenko and Rusakov, 2007
*R*_std_	Standard deviation of nanocolumn receptors distribution	nm	50	Tang et al., 2016
*D*_ani_	Diameter of nanocolumn	nm	50–500	Tang et al., 2016
*D*	Glutamate diffusion coefficient	μm^2^/ms	0.3	Budisantoso et al., 2013
*D*_r_	Receptor diffusion coefficient	μm^2^/s	0.1–1	Groc and Choquet, 2020
*g*_unit_	Unitary conductance	pS	25	Savtchenko and Rusakov, 2007
*E*_intra_	Intracellular potential	mV	65	Savtchenko et al., 2000
Res	Electrical resistivity of the medium	Ω·cm	200	Savtchenko and Rusakov, 2007
*N*_*A*_	Avogadro number	mol^−1^	6.022 × 10^23^	
*F*	Faraday constant	C/mol	96 485	
*Rg*	Perfect Gas constant	J/(mol·K)	8.3144	
*T*	Absolute temperature	K	300	
**Simulation output**				
*I*(*t*)	Mean current as a function of time *t*	pA	0–150	
*Q*	Mean total charge transfer	fC	0–100	
τ_rise_	Fitted rise time	μs	0–1,500	
τ_decay_	Fitted decay time	ms	0–2	
**Computational variables**				
Δ*t*	Time step	ns	50	
Dur	Duration of simulations	ms	0.3–250	
*b*_rad_	Binding radius	nm	5	

We show that when a presynaptic vesicle releases a large number of glutamate molecules, the presence of trans-synaptic nanocolumns has little impact on the amplitude of synaptic currents. This can be explained by the fact that when there are enough glutamate molecules to be simultaneously captured by all receptors, the presence of the nanocolumn does little to further promote the binding of glutamate molecules to receptors. On the other hand, the presence of nanocolumns increases the amplitude of synaptic currents caused by the opening of small presynaptic vesicles by increasing glutamate concentration in the vicinity of postsynaptic channels and lengthening the dwell time of these neurotransmitter molecules in the receptor dense area. Our work also reveals that the impact of nanocolumns on the strength of synaptic currents is modulated by a variety of parameters such as the cleft height and the binding affinity of glutamate molecules to AMPA receptors. For instance, when the synapse is very narrow, the presence of trans-synaptic molecules has less impact on the distribution of glutamate molecules. Also, the relative impact of nanocolumns is larger when the affinity is decreased. A third finding is that the impact of a nanocolumn is much more important when we assume that the vesicle docking is aligned with the nanocolumn than when we consider vesicle openings occurring at random locations. Finally, we investigate the impact of the lateral diffusion of postsynaptic AMPA receptor. Previous work (Choquet and Triller, 2003) showed that this lateral diffusion can mitigate receptor desensitization and thus enhance synaptic currents in scenario of repeated stimulation. While we observed this effect in our simulations, our work suggests that the presence of a nanocolumn may lessen the effect of receptor diffusion.

## 2. Materials and methods

Signal transmission between neurons can be divided into the following steps: (1) neurotransmitter molecules are released by a presynaptic vesicle either as a result of an action potential or spontaneously (Wang et al., 2016). (2) These neurotransmitter molecules diffuse across the synaptic cleft and bind to postsynaptic receptors (Barreda and Zhou, 2011; Budisantoso et al., 2013; Gerstner et al., 2014). (3) postsynaptic channels experience state transitions and eventually open resulting in transmembrane current (Koch and Segev, 1998). We model each of these steps and describe a closed-loop between the synaptic current and the diffusion of neurotransmitter molecules. On the one hand, the diffusion of neurotransmitter molecules has an obvious impact on the binding and opening of postsynaptic receptors and thus on synaptic current. On the other hand, the synaptic current creates an electric field within the synaptic cleft which can in turn influence the displacement of charged neurotransmitter molecules (such as glutamate which we simulate here; Sylantyev et al., 2008). This implies that in principle steps (2) and (3) cannot be computed separately and we take this into account. On longer time scales, the lateral diffusion of postsynaptic receptors can also play a role in modulating the amplitude of the synaptic current. Accordingly, we described the diffusion of AMPA receptors in some of our simulations.

### Modeling the diffusion of glutamate molecules

We model the synaptic cleft as a cylinder (Freche et al., 2011) with a radius of *R* = 1, 000 nm and assume in most simulations that the presynaptic vesicle releases glutamate molecules at the center top of the cleft (Savtchenko et al., 2000). The schematic of the synapse is presented in Figure 1A. We assume that at time *t* = 0 ms, all glutamate molecules are instantaneously released at the top of the synapse. The initial radial position of each of these molecules is drawn randomly according to an independent uniform distribution on a disk whose radius is equal to the radius of the vesicle *r*_ves_ = 20 nm (Clements et al., 1992; Kleinle et al., 1996). Remark that a spherical vesicle with a radius of 20 nm has a volume of ≈3.35 × 10^−23^*m*^3^. Assuming a concentration of neurotransmitter molecules in the vesicle of 50–300 mM (Scimemi and Beato, 2009), this gives a quantity of neurotransmitter molecules of ≈2 − 10 × 10^−21^ moles or about ≈1,000–6,000 molecules. For this reason, we investigate the response of the model for a number of neurotransmitter molecules in the 200–20,000 range.

We describe the motion of individual glutamate molecules by a Brownian motion (Sznitman, 2009; Mörters and Peres, 2010; Kadloor et al., 2012). At each time step and for each glutamate molecule, we choose a random unit vector uniformly distributed at the surface of a sphere. In a given time step, the molecule is moved by a distance of $\Delta r=\sqrt{6D\Delta t}$ in the direction of this random vector where *D* is the diffusion coefficient of glutamate taken as 0.3μm^2^/ms (Rice et al., 1985; Savtchenko and Rusakov, 2007) and Δ*t* is the length of the time step (Savtchenko and Rusakov, 2007). A time step of 50 ns was determined in such a way that the displacement during a single time step is in the range Δ*r* = 1 − 5 nm with Δ*t* = Δ*r*^2^/6*D*.

We validated our diffusion algorithm by simulating neurotransmitter molecule diffusion in a 3D free space. We computed the mean square displacement to verify that the model behaves as expected (see Supplementary Figure 1). The fitted value for the diffusion coefficient 0.3025 μm^2^/ms is indeed very close to the one we used to generate the simulations (0.3 μm^2^/ms).

We assume an absorbing condition at the outer edge of the synapse (Dirichlet condition; Pinchover et al., 2005), mimicking the clearance of neurotransmitter molecules by astrocytes surrounding the synapse. This implies that when a glutamate molecule reaches the outer boundary of the cylinder, it is removed from the simulation. We also consider a reflexive condition (Neumann; Pinchover et al., 2005) at the presynaptic boundary, explicitly when after moving, a glutamate molecule would have a position with a negative *z* coordinate, we replace this coordinate by |*z*|. At the postsynaptic boundary, glutamate molecules in the vicinity of a receptor (5 nm or less from the center of the receptor) have a non-zero probability to bind to this receptor. Receptors are known to protrude about 5 nm above the membrane and have a diameter of about 7 nm (Ventriglia, 2011). We have not explicitly modeled their geometry. Alternatively, when the glutamate molecule is in the space normally occupied by the receptor, we consider that it has a positive probability to bind to that receptor. In this event, the glutamate molecule becomes mobile again only when unbinding occurs. Otherwise, we use a reflexive condition (Neumann) at the postsynaptic boundary. If after moving, the glutamate molecule has a position with *z* coordinate *z*>*H*_c_ where *H*_c_ is the height of the synaptic cleft, we replace this by 2*H*_c_−*z*. In some simulations, we consider a non homogeneous and non isotropic diffusion coefficient to account for the possibility of hindered diffusion in the nanocolumn. Due to the filament shape of the trans-synaptic molecules, we assume that when they hinder diffusion, diffusion then occurs anisotropically. In this case, diffusion occurs normally in the *z* axis (perpendicular to the presynaptic boundary) but is hindered in the *xy* plane parallel to the presynaptic boundary. We use the anisotropy coefficient at the nanocolumn location (ani) as a parameter describing synaptic crowding (Zheng et al., 2017). In a previous work (Ventriglia, 2011), individual filaments were modeled with cylinders with main axis aligned with the *z* axis. The presence of regular spaced obstacle has the effective impact of slowing diffusion. Our anisotropy coefficient thus corresponds to the extent to which the trans-synaptic filaments fill the synaptic cleft. Theoretical works relate the fraction of the space occupied by regularly spaced circular (or cylindrical) obstacles to the reduction in effective diffusion coefficient. Let σ be the fraction of the space occupied by obstacles, when σ is small, the effective diffusion is reduced by a factor 1−σ as when the fraction is large, the effective diffusion is reduced by the factor [2(*π*/4−σ)^1/2^]/[*π*^3/2^(1−*π*/4)] (Farah et al., 2020). The value of the parameter describing the reduction in effective diffusion ranges from ani = 0 for totally free diffusion (no trans-synaptic filament) to ani = 1 for only “vertical” diffusion. The maximal anisotropy coefficient used in this work is 0.9, corresponding to a reduction of the diffusion in the *xy* plane of 90%, would require to a filling fraction σ≈0.78. In the absence of a nanocolumn or outside of it, the diffusion is assumed to be free and hence isotropic. Measurement of instantaneous diffusion coefficient within the synaptic cleft has also shown that nanocolumns can slow the diffusion of neurotransmitters molecules by up to 60% (Zheng et al., 2017).

### Modeling postsynaptic channels

The number of postsynaptic receptors is varied from simulation to simulation in the range 0 − 80. The location of receptors is chosen randomly in the following way. We divide the AMPA receptors into two groups, the receptors which are inside the nanocolumn (*nanocolumn receptors*) and the ones which are randomly distributed outside the nanocolumn (*randomly distributed receptors*). The number of receptors in each category (*n*_c_ and *n*_r_, respectively) is varied from simulation to simulation in the range 0 − 80. To take into account the physical size of the receptors which have a reported diameter of around 7 nm (Ventriglia, 2011), the minimum center distance between two neighbor receptors is set to 10 nm. Receptors outside the nanocolumn are distributed uniformly in the postsynaptic density (PSD) while nanocolumn receptors (or bound receptors) are distributed according to a normal distribution whose center is aligned with the center of the nanocolumn. Explicitly, the location of the nanocolumn receptors is chosen randomly in such a way that the distance between the receptor and the synapse center is given by −*R*_std_·log(1 − rand) where rand is a random variable drawn uniformly in [0, 1) and *R*_std_ is the standard deviation of the two-dimensional Gaussian. The radial position of the channels is chosen uniformly in [0, 2π). The position of receptors lying < 10 nm of another are redrawn until we get a distribution such that the center of any two given receptor is at least 10 nm for each other.

Receptor distribution is illustrated in Figure 1B. The PSD is defined as a disk at *z* = *H*_c_ centered at (*x, y*) = (0, 0), its diameter is denoted by *D*_*dr*_ and set to 400 nm in all simulations (Kleinle et al., 1996; Savtchenko et al., 2000; Franks et al., 2002). In most simulations, receptors are assumed to be immobile for the duration of the simulation which is justified by the relatively short simulated time within 3 ms. In order to investigate the impact of the lateral diffusion of postsynaptic receptors, we also described this diffusion in some simulations as described below.

Transitions between channel states are described through a discrete Markov process. We consider the nine possible receptor states illustrated in Figure 1C (Wadiche and Jahr, 2001; Raghavachari and Lisman, 2004; Scimemi and Beato, 2009). At the beginning of the simulation, all the channels are in the closed state C0 with no bound glutamate molecule. The channels require two bound glutamate molecules to open (Holmes, 1995; Santucci and Raghavachari, 2008) and thus have two additional closed states (C1 and C2) with one bound glutamate molecule and two bound glutamate molecules respectively. Other channel states include one open state (O2), one desensitized state with one bound glutamate molecule (D1) and four desensitized states with two bound glutamate molecules (D2a–D2d; Wadiche and Jahr, 2001; Scimemi and Beato, 2009). We use the kinetic scheme given in Kleinle et al. (1996), Koike et al. (2000), Franks et al. (2002), Budisantoso et al. (2013) and shown in Figure 1C.

In some simulations, we also described the lateral diffusion of postsynaptic AMPA receptors. To do this, we divided the surface of the postsynaptic space into a grid of 10 nm × 10 nm elements where 10 nm is assumed to be the size of the receptor. We assumed that a spatial element can be occupied by at most one receptor. To describe the diffusion of postsynaptic receptor we used a value of lateral diffusion coefficient of 0.1 μm^2^/s (or 1 μm^2^/s in some simulations) consistent with Groc and Choquet (2020). We computed the probability that a receptor moves to a neighbor grid element during a given time step. So at each time step and for each receptor, we first draw a random number to determine if the receptor indeed could move to a neighbor grid element. If it is the case, we then draw a random number between 1 and 4 to determine the direction (left, right, up, or down) in which the receptor would move. Finally, the receptor actually moves only if the grid element to which it would move is free (i.e., is not already occupied by a receptor). When the movement of a receptor transports it outside of the synapse, it is replaced by another receptor appearing randomly at the edge of the synapse. The new receptor is then assumed to be in state C0, that is closed with no neurotransmitter molecule attached.

### Modeling the binding of glutamate molecules to AMPA receptors

We avoid a geometrical description of individual AMPA receptor and simply specify it's position by a vector (*x, y*). We assume that a glutamate molecule has a positive probability to bind to an available AMPA receptor if it is within the *binding radius* of this receptor. This radius (5 nm) corresponds more or less to the physical extent of the receptor. Our strategy is thus equivalent to saying that there is a positive probability of binding if the glutamate molecule is in physical contact with the AMPA receptor. Our approach is thus compatible with the one used in Ventriglia (2011). In order to be able to use the binding rates which are given in the dimension of probability per time per concentration, we need to convert the presence of a glutamate molecule within the binding radius of an AMPA receptor into a concentration equivalent. We do so according to the formula $C\sim \frac{1}{N_{A}\text{Vol}}$ where *C* is the equivalent concentration and *N*_*A*_ is the Avogadro number (Taylor, 2009). Here $\text{Vol}=\frac{2\pi b_{rad}^{3}}{3}$ is the volume of a half sphere of radius *b*_*rad*_ equal to the binding radius. A bound glutamate molecule doesn't move until it unbinds.

### Modeling synaptic current and electric field

Trans-synaptic current creates an electric field within the synaptic cleft which can influence both the amplitude of the trans-synaptic current and the diffusion of charged neurotransmitter molecules within the synapse. Given this importance, we model the electric potential within the synaptic cleft. This is often overlooked as the extracellular potential is in many models taken to be constant and equal to 0 mV. However, theoretical studies (Savtchenko and Rusakov, 2007) argue that the extracellular potential inside the cleft should become hyperpolarized by a few millivolts during a synaptic event. As in Savtchenko and Rusakov (2007), we neglected the capacitive currents which is justified by the small spatial size of the cleft. By Kirchoff's law, we can obtain the electric potential within the synapse by solving the equation


(1) $$\begin{array}{l} \text{Δ}v\left(x,y,t\right)=I\left(x,y,t\right). \end{array}$$

Here *v*(*x, y, t*) is the electric potential at position *x, y* in the cleft and at time *t* and *I*(*x, y, t*) is the trans-synaptic current at position *x, y* and at time *t*. We used the Dirichlet boundary condition that the potential is equal to 0 mV at the outer edge of the cleft and the initial condition that the potential is equal to 0 mV at the beginning of the simulation.

In order to compute the electric field (i.e., to solve Equation 1), we used a finite volume approach. That is, we subdivide the synaptic cleft into a grid of parallelepiped elements with 20 nm × 20 nm squared base and with a height equal to the synaptic cleft height. At each time step, an electric potential is computed for each spatial element. We denote by *v*_*i, j*_(*t*) the electrical potential of element at coordinates *i, j* and at time *t*. The grid referred to in this paragraph is a purely computational tool used to solve a partial differential equation specifying the electric field. Extra simulations (not shown) convinced us that the size of the elements of this computational grid is not critical. The element size of 20 nm was chosen as a compromise between accuracy and computational cost. The intracellular potential evolves during a synaptic event as is described by many classical models. For synapses located on dendritic spines, the extent of the depolarization during an excitatory event is largely dependent on the resistance of the spine neck which itself depends on spine geometry. For synapses located on a spine with a short and thick neck, the depolarization occurring in the spine can be as small as 1–2 mV. Given this, given the great variability in spine geometry and given that the focus of this work is not a full description of the postsynaptic neuron, we kept the intracellular potential constant and equal to the resting value of −65 mV (Li et al., 2018). We refer to the intracellular potential as (*E*_intra_) to avoid confusion with the extracellular potential (in the cleft) which is considered as a dynamical variable in our simulations.

The reversal potential of the synapse or synaptic potential (*E*_*syn*_) is the value of the membrane potential for which there is no net current through the synaptic channels when they are open. The synaptic potential is determined by the concentrations of the various ionic species mediating the synaptic current and can be computed by the Goldman Hodgkin Katz equation. For synapses with AMPA receptors, the current is mediated by sodium and potassium ions and we have *E*_*syn*_≈0. In this work, for the sake of simplicity, we use the value *E*_*syn*_ = 0*mV*. The current through a synapse is classically given by the equation


(2) $$\begin{array}{l} I_{syn}\left(t\right)=g_{syn}\left(t\right)\left(E_{syn}-V_{m}\left(t\right)\right) \end{array}$$

with the convention that a positive current is depolarizing. The synaptic conductance *g*_*syn*_(*t*) can be written as *g*_*syn*_(*t*) = *n*(*t*) × *g*_*unit*_ where *n*(*t*) is the number of open channels and *g*_*unit*_ is the unitary conductance of a single open channel. In Equation (2), *V*_*m*_(*t*) is the membrane potential which is by definition equal to the difference between the intracellular and extracellular potential. When the extracellular potential is taken as constant and equal to 0 mV, the membrane potential is simply equal to the intracellular potential. However, in our model, we consider the intracellular potential to be constant (*E*_*intra*_ = −65 mV) and the extracellular potential in the cleft to be dynamic *v*(*t*), the membrane potential will instead by given by *V*_*m*_(*t*) = *E*_*intra*_−*v*(*t*).

It follows from Equation (2) that the transmembrane synaptic current through a spatial element of coordinates *i, j* at time *t* is given by (Savtchenko et al., 2017)


$$I_{i,j}\left(t\right)=n_{i,j}\left(t\right)\times g_{\text{unit}}\times \left(v_{i,j}\left(t\right)-E_{\text{intra}}\right).$$

Here, *n*_*i, j*_(*t*) is the number of open channels in the element of coordinates *i, j* at time *t*, *g*_unit_ is the unitary channel conductance set at 25 pS (Savtchenko and Rusakov, 2007), *E*_intra_ is the fixed intracellular potential set at −65 mV (Savtchenko et al., 2000) and *v*_*i, j*_(*t*) is the electric potential in the synaptic cleft at spatial element *i, j* and at time *t*. In order to compute the electric potential *v*_*i, j*_(*t*), we neglect the capacitive current and thus assume that the net current through each spatial element at each time point is equal to zero. The current between two adjacent spatial elements (say at coordinates *i, j* and coordinates *i*−1, *j*) is given by


$$\left(v_{i,j}\left(t\right)-v_{i-1,j}\left(t\right)\right)\times H_{\text{c}}/\text{Res}$$

where *H*_c_ is the height of the synaptic cleft and Res is the electrical resistivity of the extracellular medium (200 Ω·cm; Savtchenko and Rusakov, 2007). Thus, the electric potential of each element is obtained by solving the following equation


(3) $$\begin{array}{l} \;n_{i,j}(t)\times g_{\text{unit}}\times (v_{i,j}(t)-E_{\text{intra}})\\ =-\left[4v_{i,j}(t)-v_{i-1,j}(t)-v_{i+1,j}(t)-v_{i,j-1}(t)-v_{i,j+1}(t)\right]\\ \times \frac{H_{\text{c}}}{\text{Res}}. \end{array}$$

Equation (3) is a discretized version of Equation (1). It yields a system of linear equations with respect to the vector $\vec{v}\left(t\right)=\left(v_{1,1}\left(t\right),\ldots ,v_{1,n}\left(t\right),v_{2,1}\left(t\right),\ldots ,v_{n,n}\left(t\right)\right)$ describing the electric potential within the cleft. This system can be written as $A\left(t\right)\vec{v}\left(t\right)=\vec{b}\left(t\right)$, where *A*(*t*) is a square matrix whose entries are function of the number of open channels in each spatial element *n*_*i, j*_(*t*) and $\vec{b}\left(t\right)$ is a constant vector whose entries are also a function of *n*_*i, j*_(*t*).

We solve this vectorial equation at every time step to deduce the electric potential in the synapse. The electric gradients along the *x* and *y* axes are, respectively given by


(4) $$\begin{array}{l} \Delta v_{x,i,j}(t)=\frac{v_{i+1,j}(t)-v_{i-1,j}(t)}{2d}\text{ and }\Delta v_{y,i,j}(t)\\ \;=\frac{v_{i,j+1}(t)-v_{i,j-1}(t)}{2d} \end{array}$$

where *d* is the size of a spatial element (20 nm). The position of a glutamate molecule at each time step is thus updated as in Sylantyev et al. (2008) according to


(5) $$\begin{array}{l} \vec{r}(t+\Delta t)=\vec{r}(t)+\sqrt{6D\Delta t}\times \vec{u}\cdot (1-\text{ani},1-\text{ani},1)+\\ \;\Delta t\times ((1-\text{ani})\times \Delta v_{x,i,j}(t),(1-\text{ani})\\ \;\times \;\Delta v_{y,i,j},0)\frac{DF}{RgT}. \end{array}$$

Here, $\vec{r}$ stands for the position vector of a given glutamate molecule, ani stands for the anisotropy coefficient, *F* for the Faraday constant, *Rg* for the perfect gas constant and *T* for the absolute temperature. In Equation (5), $\vec{u}$ is a unit vector uniformly drawn at the surface of a sphere and · stands for a component per component vector product. The second term of the right-hand side in Equation (5) describes the random part of the glutamate molecule displacement due to the Brownian motion while the third term is due to the impact of the electric field on the charged glutamate molecule (valence = −1; Savtchenko et al., 2000; Savtchenko and Rusakov, 2007).

It could have been interesting to add other elements to our model such as a description of the postsynaptic or presynaptic potential or a description of neurotransmitters buffering by neighbor astrocytes. However, we feel that these additions would be beyond the scope of the present work.

### Numerical implementation

At time *t* = 0 in our simulations, a presynaptic vesicle opens and the initial position of glutamate molecules satisfy *z* = 0. Their initial position in the *xy* plane is chosen randomly from a uniform distribution on a disk corresponding to the surface of the vesicle (*r*_ves_ = 20 nm). The simulation time was divided into equal time steps of length Δ*t* = 50*ns*. This value was chosen so that the displacement of a glutamate molecule during a time step is not larger that the physical extent of an AMPA receptor. At each time step, the direction of the random component of each glutamate molecule displacement was chosen as a unitary vector $\vec{u}=\left(x,y,z\right)$ uniformly drawn from the surface of a sphere. Specifically, we draw two random numbers *a* and *b* uniformly and independently between 0 and 1 and the unitary random direction vector is given by $\vec{u}=\left(\sin\left(\phi \right)\cos\left(\theta \right),\sin\left(\phi \right)\sin\left(\theta \right),\cos\left(\phi \right)\right)$, where θ = 2π*a* and ϕ = arccos(1 − 2*b*). When the final position of the glutamate molecule satisfies *z* < 0, we assume a reflexive condition (Neumann condition). Explicitly, if the final *z* position is *z* = −ε, (ε>0) we change it for *z* = ε. A glutamate molecule is considered absorbed and removed from play (Dirichlet condition), when it's end position in the *xy* plane satisfies *x*^2^+*y*^2^≥*R*^2^. When the end position of the neurotransmitter molecule passes the postsynaptic membrane, the *z* position is changed from *H*_c_+ε to *H*_c_−ε where *H*_c_ is the height of the synaptic cleft. We also model the binding of glutamate molecules by the receptors. To do so, we define a capture radius of 5 nm so that the neurotransmitter molecule has a positive probability of being captured by the receptor if the distance between the receptor and the neurotransmitter molecule is less than the capture radius. The probability of being captured in the event of a neurotransmitter molecule being close enough to a receptor is given by *p*_*capt*_ = Δ*t* × *C*, where *C* is the equivalent concentration defined earlier. The displacement and capture of neurotransmitter molecule is implemented in a vectorial manner in MATLAB.

Our model contains many stochastic steps related to the displacement of glutamate molecules, the lateral diffusion of AMPA receptors and the transition between channel states. We implement a Monte Carlo approach and results are taken as the average taken over many simulations. On the one hand, in order to obtain reasonably smooth averaged time traces, we needed to a large number of simulations as individual realizations are very noisy. On the other hand, the computation cost increases with the number of repetitions as well as with the number of glutamate molecules considered in the model. The number of repetitions (1, 000, 000/nnt) was chosen as a compromise between these two constraints and was in any case in the 50–1,000 range. The computational cost of simulations increased with the number of glutamate molecules in an almost linear fashion. So, in order to strike a balance between the numerical accuracy and the computational time require, we decrease the number of trials when the simulations involved a large number of glutamate molecules. In some simulations, we described the lateral diffusion of AMPA receptors. In these simulations, we modeled trains of simulations lasting from 50 to 250 ms depending on the stimulation frequency. Given the long duration of these simulations, we only performed 20 repetitions in these cases.

In the grouped analysis of many simulations, we extract the rise time and decay time from the mean synaptic currents averaged over many stochastic realizations of the same simulated scenario. To extract these values, we fit the function (Major et al., 1994; Hardingham et al., 2010).


(6) $$\tilde{I}\left(t\right)=\frac{Q}{\tau_{\text{decay}}-\tau_{\text{rise}}}\left(e^{-t/\tau_{\text{decay}}}-e^{-t/\tau_{\text{rise}}}\right),$$

to the mean of the simulated current *I*(*t*). Remark that τ_*decay*_≫τ_*rise*_ and that Equation (6) would return a positive current whether τ_rise_ < τ_decay_ or τ_rise_ > τ_decay_. In fact, the two time constants (τ_*rise*_ and τ_*decay*_) are interchangeable in Equation (6). The parameters *Q*, τ_*rise*_ and τ_*decay*_, with the constraint that τ_*rise*_ < τ_*decay*_, are chosen as to minimize the mean square error ∫(Ĩ(*t*)−*I*(*t*))^2^*dt* where the time integral is taken over the duration of the simulation. In Equation (6), *Q* is the total charge passing through the synapse, τ_rise_ and τ_decay_ are the rise time and decay time, respectively. Table 1 outlines the values, or range of values, of the parameters used in our simulations.

## 3. Results

**Figure 2:**
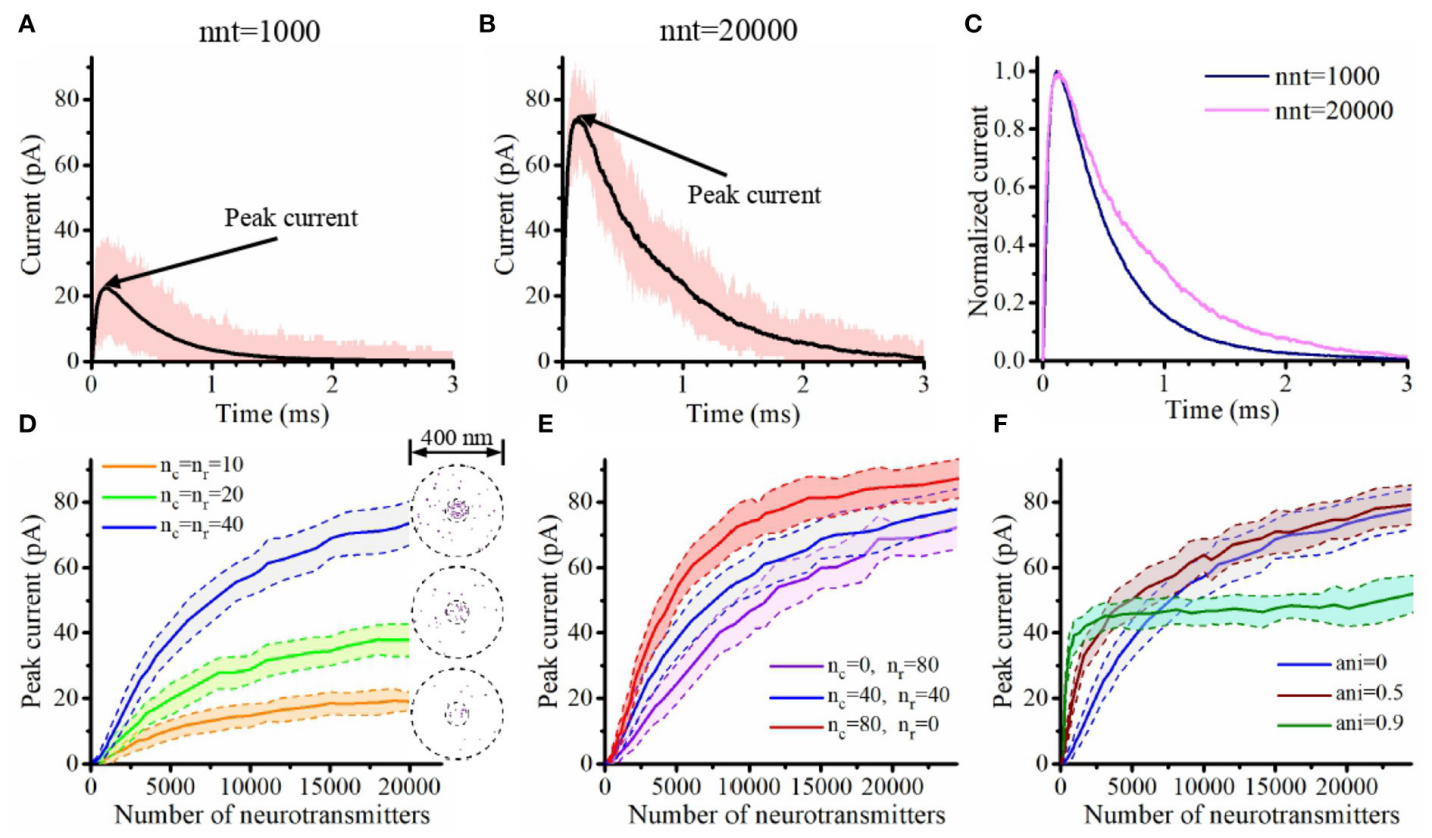
Time course and maximal amplitude of synaptic current. **(A,B)** Time course of synaptic current with nnt = 1, 000 **(A)** and nnt = 20, 000 **(B)**. Simulations were performed with the following parameters *n*_c_ = *n*_r_ = 40, ani = 0.5, *D*_ani_ = 100 nm, *R*_std_ = 50 nm, and *H*_c_ = 20 nm. The black curves correspond to the mean value taken over 1, 000, 000/nnt repetitions while the pink shadow correspond to the range of the peak current during the 1, 000, 000/nnt repetitions. The rise time, decay time and total charge transfer are, respectively τ_rise_ = 53 μs, τ_decay_ = 0.45 ms and *Q* = 13 fC for **(A)** and τ_rise_ = 39 μs, τ_decay_ = 0.71 ms and *Q* = 62 fC for **(B)**. **(C)** Normalized mean current. **(D)** Peak current as a function of the number of released glutamate molecules when *n*_c_ = *n*_r_ = 10, *n*_c_ = *n*_r_ = 20, and *n*_c_ = *n*_r_ = 40, respectively. Right of each curve, we display an example of random channel distribution. Here, we set ani = 0, *D*_ani_ = 100 nm, *R*_std_ = 50 nm, and *H*_c_ = 20 nm. **(E)** Peak current as a function of the number of released glutamate molecules when *n*_c_ = 0, *n*_r_ = 80, *n*_c_ = *n*_r_ = 40, and *n*_c_ = 80, *n*_r_ = 0. Here, we set ani = 0, *D*_ani_ = 100 nm, *R*_std_ = 50 nm, and *H*_c_ = 20 nm. **(F)** Peak current as a function of number of released glutamate molecules when ani = 0, ani = 0.5 and ani = 0.9. Here, we set *n*_c_ = *n*_r_ = 40, *D*_ani_ = 100 nm, *R*_std_ = 50 nm, and *H*_c_ = 20 nm. The shaded areas in **(D–F)** represent the range of the peak current during 50 simulations (± one standard deviation).

Using our innovative mathematical model based on a Monte Carlo approach, we investigate the impact of parameters that define the organization of the trans-synaptic nanocolumn on synaptic current. These parameters include the numbers of receptors inside and outside the nanocolumn, the alignment between the receptors and the release site and the extent to which trans-synaptic filaments reduce the effective diffusion coefficient of glutamate molecules. We also investigated how the presence of a nanocolumn modulates the impact of postsynaptic receptor diffusion on the synaptic response. A preliminary step was to verify that our model could replicate the typical time course of synaptic currents and that nanocolumns could indeed impact the amplitude and temporal features of synaptic currents. We started by performing two simulations with a number of released glutamate molecules (1,000 and 20,000) at the lower and upper ends of the plausible range (Savtchenko and Rusakov, 2007; Budisantoso et al., 2013; Figure 2). As discussed in Section 2, a spherical presynaptic vesicle of 20 nm radius with a neurotransmitter molecule concentration of 200 mM would contain about 3,000 neurotransmitter molecules and the value of 6,000 neurotransmitter molecules can be found in the literature (Budisantoso et al., 2013). We choose to cover a wide interval around these typical values taking advantage of the flexible of an *in silico* approach. In both cases (nnt = 1, 000 and nnt = 20, 000), we observed the typical rapid rise and slower decay of synaptic currents (Figures 2A,B). The red curves illustrate a single simulation highlighting the stochastic nature of our model while the black curves are averages taken over 1,000 and 50 simulations, respectively. Normalizing the mean current curves Figure 2C shows that the decay time constant was larger when 20,000 glutamate molecules were used. Next, we tested how peak current depends on the number of released glutamate molecules. For this, we used different numbers of AMPA receptors but always in a scenario where half of the AMPA receptors are inside of the nanocolumn and half are outside of it, as illustrated in Figure 2D. For every number of AMPA receptors investigated, we observe a similar tendency. We see a linear increase of the peak currents as a function of the number of released glutamate molecules when this number is relatively low. However, the peak currents reach a plateau when the number of glutamate molecules is larger. This is explained by the fact that when the number of glutamate concentration is large enough so that every receptor is occupied, further increasing the number of released glutamate molecules will not further increase the probability of binding. To test if the presence of a nanocolumn could significantly impact trans-synaptic currents, we investigated scenarios with different proportions of channels associated to the nanocolumn and different hindrances to diffusion (anisotropy coefficients) as shown in Figures 2E,F. Beyond confirming the importance of the nanocolumns, these results guided our subsequent investigations in which we mostly use a number of glutamate molecules for which the impact of a nanocolumn is likely to be significant (i.e., nnt between 1,000 and 10,000).

### Typical time course of glutamate binding and receptor state transition

**Figure 3:**
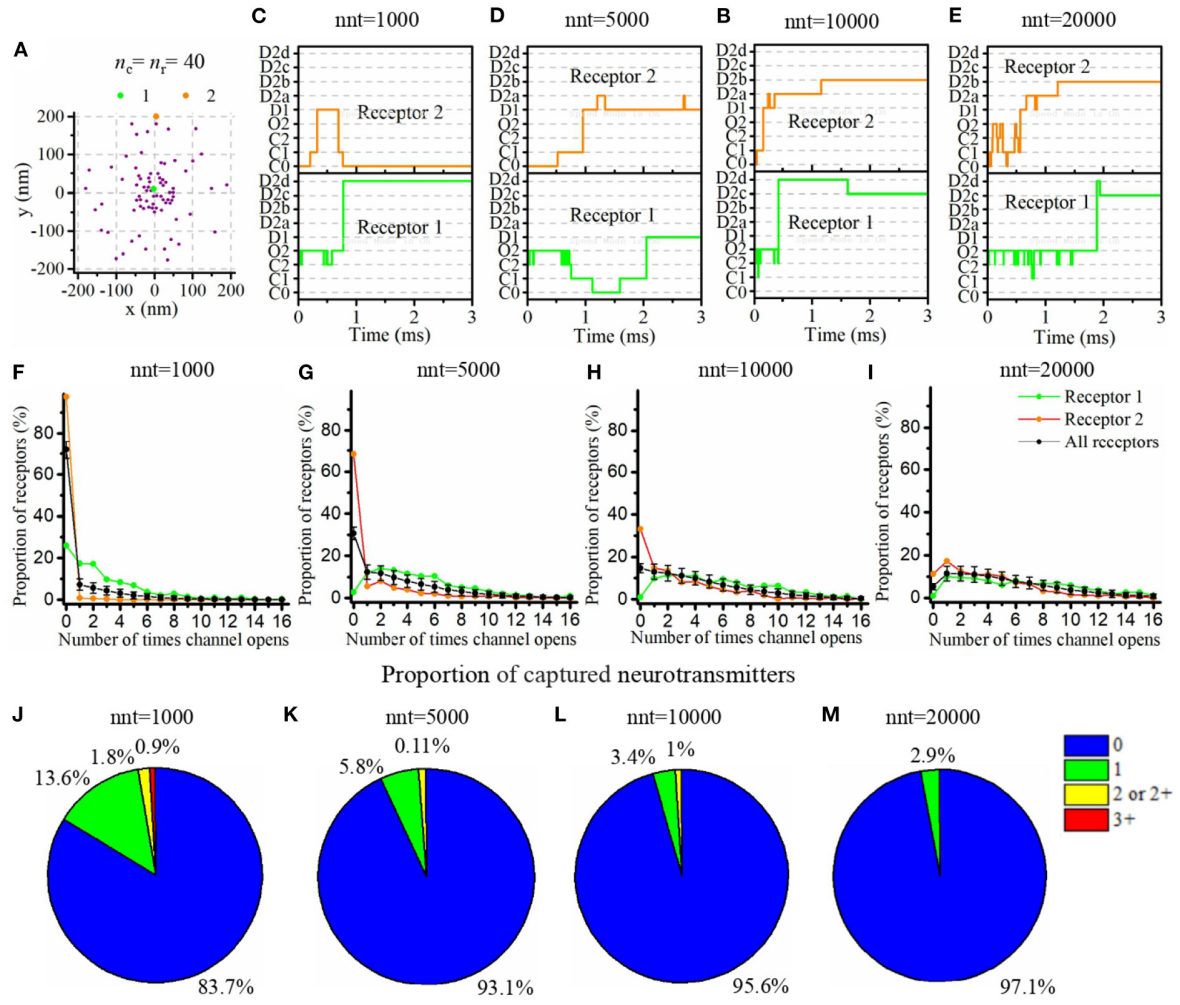
Time course of AMPA receptor state transition and glutamate binding. **(A)** Random distribution of receptors with *n*_c_ = *n*_r_ = 40. We pick two receptors (marked as 1, 2, and located approximately at 0 and 200 nm from the synapse center) and record the temporal evolution of their state for different numbers of released glutamate molecules [**(B)** nnt = 1, 000, **(C)** nnt = 5, 000, **(D)** nnt = 10, 000, and **(E)** nnt = 20, 000]. A single simulation is shown in each case for the sake of illustration. **(F–I)** The number of times receptors 1 and 2 open taken over 500 simulations. The black line corresponds to the average taken over all receptors and the black bars represent the standard deviation. **(J–M)** We also tracked individual glutamate molecules and monitored the number of times each one binds to a receptor. We conducted simulations with 1,000, 5,000, 10,000, and 20,000 released glutamate molecules. When only 1,000 glutamate molecules were released **(J)**, about 16% bounded at least once to a receptor and 3% twice or more. These proportions only slightly decreased when 5,000 glutamate molecules were released **(K)**. We observe a noticeable decrease in this proportion for 10,000 glutamate molecules **(L)** and for 20,000 glutamate molecules, **(M)** almost all of them were never captured. For all cases, *D*_ani_ = 100 nm, *R*_std_ = 50 nm, ani = 0.5, and *H*_*c*_ = 20 nm.

Our mathematical model allows to track the individual states of each AMPA receptor (Figure 1C) as well as the individual location of each glutamate molecule at each time step. We took advantage of this and investigated typical examples of channel responses in Figure 3. The typical time course of state transitions for a given receptor is expected to depend heavily on its location. Indeed, channels located at the center of synapse and inside of the nanocolumn are more likely to open than those located outside of the nanocolumn. Because of this, we focused our attention on two receptors, one at the center of the synapse and at the center of the nanocolumn (green) and one at 200 nm from it (orange) (see Figure 3A). Sample time courses of the states of these receptors are given in Figures 3B–E. We see that, as can be expected from the rate constants, receptors can remain desensitized for long periods of times, (see Receptor 1 in Figure 3B and Receptor 2 in Figure 3D) while rapid oscillations between open and close states are also possible (see Receptor 1 in Figures 3D,E). On longer time scales (hundreds of milliseconds) channels states would eventually evolve toward the state C0, that is the close and unbound state which is the only absorbing state of the Markov process in the absence of neurotransmitter molecules. Because they will sense a greater glutamate concentration, receptors located right under the vesicle (at the center of the synapse in this example) are expected to be open longer which is indeed observed in Figures 3F–I. In this set of simulations, we use 40 receptors located inside of the nanocolumn and 40 receptors randomly distributed outside of it. The standard deviation of the spatial distribution of the nanocolumn receptors is 50 nm. We assume that the anisotropy of the coefficient of diffusion under the synaptic cleft is equal to 0.5. In other words, we assume the crowding of the synaptic cleft reduces the longitudinal diffusion coefficient of glutamate molecules by half within the nanocolumn where trans-synaptic filaments are present.

The time course of receptor state transition is also heavily dependant on the number of released glutamate molecules. To investigate this dependency, we repeated the simulation with different numbers of released glutamate molecules (1,000, 5,000, 10,000, and 20,000) and, for each of these values, we computed the distribution of the number of channel openings. We found that, when only 1,000 glutamate molecules are released, 72% channels never go into open state. This proportion falls to 31% as soon as 5,000 glutamate molecules are considered. At the other end of the spectrum, when 20,000 glutamate molecules are released, 9.5% of all channels open 12 times or more (see Figures 3F–I). This large number of transitions into open state is mainly due to rapid back and forth between closed bound state and open state (see Supplementary Figure 2). We also took advantage of the fact that our modeling approach tracks individual glutamate molecules to compute the number of times each glutamate molecule binds to an AMPA receptor. Many models rely on a continuous approximation to describe the concentration neurotransmitter molecules. The correctness of such an approach relies on the assumption that the fraction of neurotransmitter molecules that are captured and released by the receptors can be neglected. In other words, most modeling approaches assume that the time course of the concentration of neurotransmitter molecules is not impacted by the binding and unbinding of these molecules to the postsynaptic receptors. In Figures 3J–M, we show the distribution of the number of captures of each glutamate molecule. This reveals that for a large number of glutamate molecules (nnt = 20, 000; Figure 3M), the proportion of these molecules that never bind to a receptor is close to 1. Hence, in this scenario, the use of a continuous approximation for the concentration would not be expected to alter significantly the results. However, on the other extreme, when a very small number of glutamate molecules is considered (nnt = 1, 000; Figure 3J), the proportion of glutamate molecules that are captured at least once becomes about 16% while a significant proportion of glutamate molecules are captured twice or more. From a methodological point of view, this suggests that for small numbers of released glutamate molecules, it becomes important to track the individual binding of these molecules. The continuous concentration approximation might be misleading in this case. The binding and unbinding of glutamate molecules prolongs their dwelling time in the synaptic cleft which must be taken into account. To further investigate this, we fixed a small number of released glutamate molecules (nnt=500) and we compared three modeling approaches: (1) The main model used in this manuscript and described above, (2) A model in which captured glutamate molecules are removed from the simulation, (3) A model in which the capture of a glutamate molecule doesn't affect the glutamate concentration (i.e., the channels is labeled as bound but the glutamate molecule keeps moving). The chosen scenario can have an impact on the kinetics of the synaptic current (Supplementary Figure 3). It can thus be important to accurately describe the binding and unbinding of neurotransmitter molecules as we did in the present work even if it is more computationally expensive.

Having validated our modeling approach, we move to study in more details the impact of the parameters characterizing the nanocolumn properties in our model on the features of synaptic currents.

### Trans-synaptic filaments slowing glutamate diffusion

To investigate the functional impact of nanocolumns, we describe their presence and importance with the following parameters: (1) ani. The anisotropy coefficient describing how trans-synaptic molecules impair glutamate diffusion in the plane parallel to the presynaptic membrane. We thus consider the homogenized effect of the trans-synaptic filaments instead of describing the geometry of individual of each filament as it was done in Ventriglia (2011). (2) *n*_c_, *n*_r_. The number of receptors inside and outside of the nanocolumn. We also refer to receptors outside the nanocolumn as randomly distributed receptors. A larger *n*_c_/*n*_r_ ratio indicates a greater importance of the nanocolumn. (3) *D*_ani_. The diameter of nanocolumn or more specifically the diameter of the region in which anisotropic diffusion is assumed to occur or equivalently the region of the synaptic cleft where we assume trans-synaptic filaments to be present. We investigate the impact of these parameters on features of the synaptic current including peak current, rise time, decay time, total charge transfer as well as the proportion of neurotransmitter (glutamate) molecules that are captured at least once during the simulation, i.e., the proportion of glutamate molecules that are actually used by the synapse.

**Figure 4:**
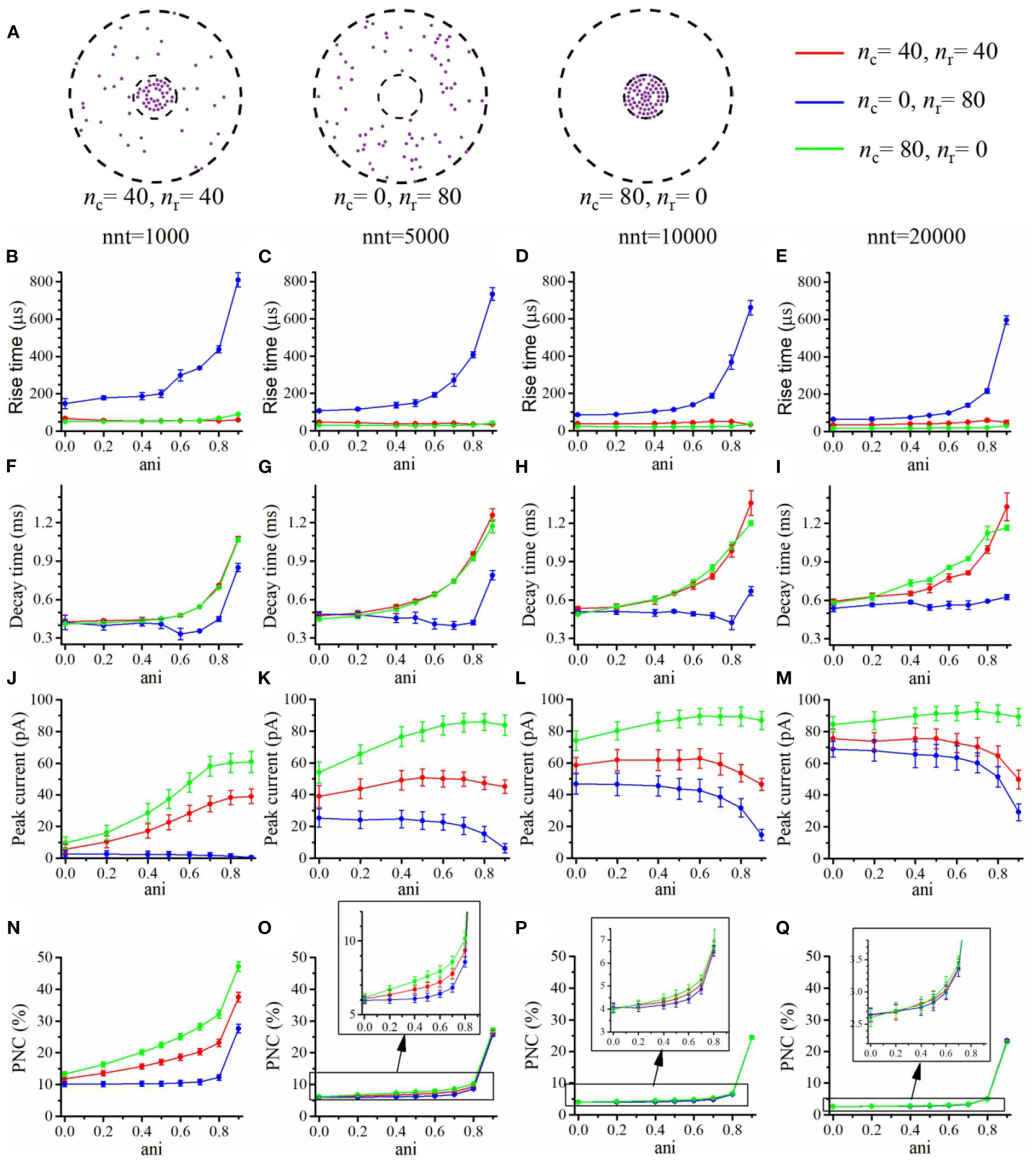
Influence of the anisotropy coefficient (synaptic crowding). **(A)** Receptor distribution for different combinations of *n*_r_ and *n*_c_. Rise time as a function of anisotropy coefficient (ani) for **(B)** nnt = 1, 000, **(C)** nnt = 5, 000, **(D)** nnt = 10, 000, and **(E)** nnt = 20, 000 released glutamate molecules. Decay time as a function of ani for **(F)** nnt = 1, 000, **(G)** nnt = 5, 000, **(H)** nnt = 10, 000 and **(I)** nnt = 20, 000. Peak current as a function of ani for **(J)** nnt = 1, 000, **(K)** nnt = 5, 000, **(L)** nnt = 10, 000, and **(M)** nnt = 20, 000. Proportion of neurotransmitter molecules captured (PNC) as a function of ani for **(N)** nnt = 1, 000, **(O)** nnt = 5, 000, **(P)** nnt = 10, 000, and **(Q)** nnt = 20, 000. The red curves are obtained with *n*_c_ = *n*_r_ = 40, the blue curves with *n*_c_ = 0, *n*_r_ = 80, and the green ones with *n*_c_ = 80, *n*_r_ = 0. The error bars mark the standard deviation. For this set of simulations, we choose *R*_std_ = 50 nm, *D*_ani_ = 100 nm, and *H*_c_ = 20 nm.

**Figure 5:**
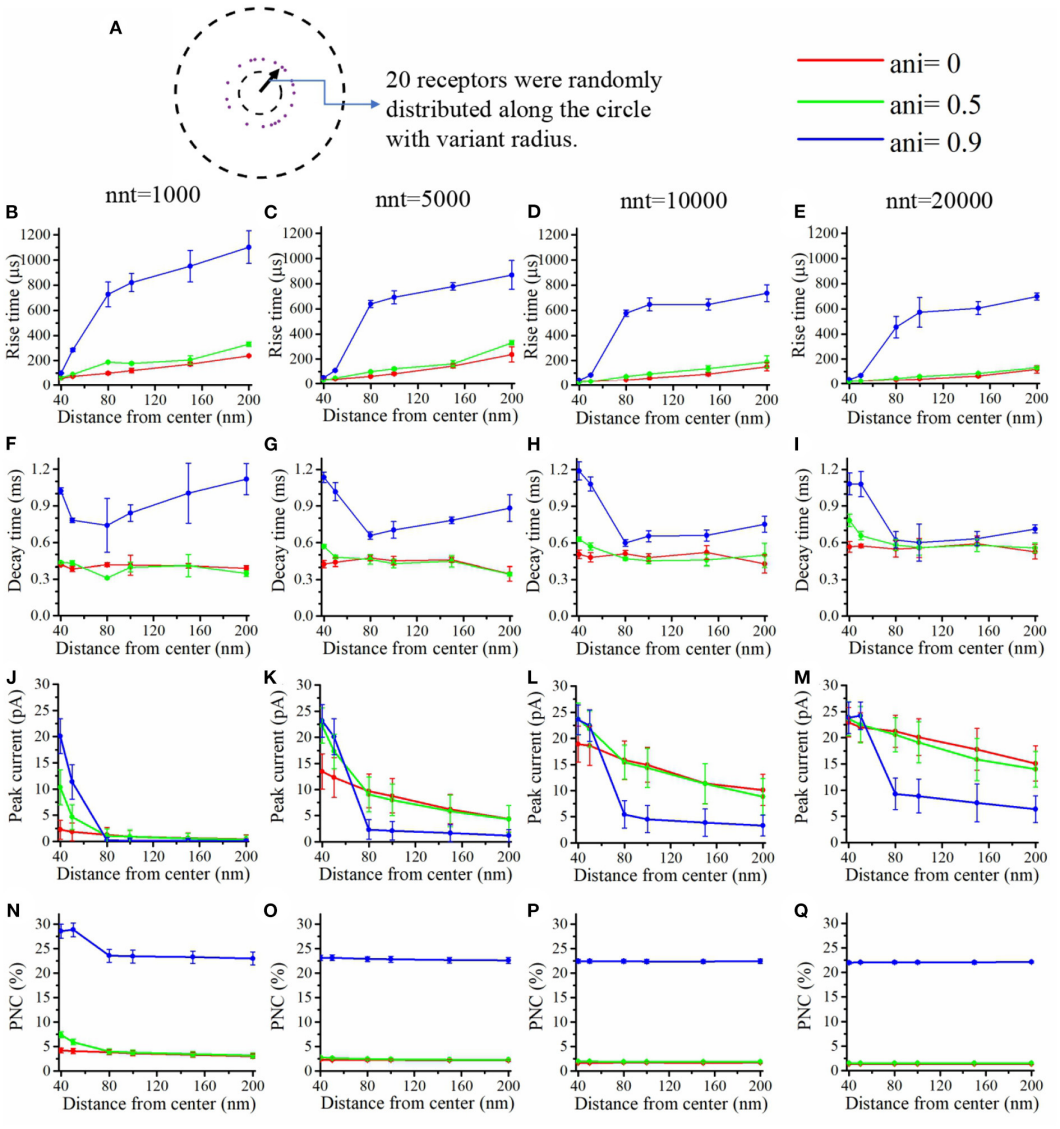
Influence of the position of the AMPA receptors in the postsynaptic zone. **(A)** In this set of simulations, 20 receptors are placed on circles with centers coinciding with the synapse center at random radial positions. Rise time as a function of receptor distance from the synapse center for **(B)** nnt = 1, 000, **(C)** nnt = 5, 000, **(D)** nnt = 10, 000, and **(E)** nnt = 20, 000 released glutamate molecules. Decay time as a function of the distance between AMPA receptors and the synapse center for **(F)** nnt = 1, 000, **(G)** nnt = 5, 000, **(H)** nnt = 10, 000, and **(I)** nnt = 20, 000 neurotransmitter molecules. Peak current as a function of the distance between AMPA receptors and the synapse center for **(J)** nnt = 1, 000, **(K)** nnt = 5, 000, **(L)** nnt = 10, 000, and **(M)** nnt = 20, 000. Proportion of neurotransmitter molecules captured (PNC) as a function of the distance between receptors and the synapse center for **(N)** nnt = 1, 000, **(O)** nnt = 5, 000, **(P)** nnt = 10, 000, and **(Q)** nnt = 20, 000. Red curves are obtained with ani = 0, green curves with ani = 0.5 and blue curves with ani = 0.9. The error bars mark the standard deviation. The mean value is taken over 1, 000, 000/nnt repetitions. Other parameters were set to *D*_ani_ = 100 nm and *H*_c_ = 20 nm.

We first focused our attention on the impact of the anisotropy coefficient (Figure 4) quantifying the synaptic cleft crowding by trans-synaptic molecules as described in the method section. A first somewhat surprising observation is that the anisotropy coefficient can either increase or decrease in the peak amplitude of synaptic current depending on the values of other parameters. In all simulations, we assumed that the total number of receptors is equal to 80. We considered a mixed scenario (red curves) in which there are 40 receptors placed inside the nanocolumn 40 receptors placed outside the nanocolumn, as well as a scenario in which all receptors are inside the nanocolumn (green curves) and one in which all receptors are outside of the (blue curves). In the scenario where all receptors are inside the nanocolumn (Figures 4J–M), increasing the anisotropy coefficient always leads to an increase in peak current. This increase is especially important when the number of released glutamate molecules is small (1,000 or 5,000) while this effect is less important when considering a release of 20,000 glutamate molecules. In the latter case, most channels will capture glutamate molecules no matter how trans-synaptic filaments impact their diffusion. Molecules crowding the synapse in the nanocolumn area prolong the dwelling time of glutamate molecules in the vicinity of receptors lying right under the vesicle which leads to an increase in peak current if a large proportion of receptors are inside the nanocolumn. On the other end, when we consider channels lying mostly outside of the zone where trans-synaptic filaments are assumed to be present, the peak current decreases for very large values of anisotropy coefficients. This is explained by the fact that in this scenario, a large anisotropy coefficient (i.e., a scenario where filaments crowding the synaptic cleft hinder diffusion) delays the contact between the glutamate molecules and AMPA receptors. We also investigated the impact of the anisotropy coefficient on the proportion of neurotransmitter (glutamate) molecules which bind at least once to a receptor. This, in a sense, corresponds to the proportion of glutamate molecules which are useful. We can observe (Figures 4N–Q) that when all receptors are inside the nanocolumn, increasing the anisotropy coefficient leads to an increase in the proportion of “useful” glutamate molecules. The effect was less significant when considering a mixed scenario or a scenario in which all receptors are located outside of the nanocolumn. Finally, we investigated the impact of the anisotropy coefficient on the rise time and decay time of synaptic currents. This impact on the rise time (Figures 4B–E) was small except for very large anisotropy coefficients (>0.7). With respect to decay time (Figures 4F–I), we observed that increasing the anisotropy coefficient led to an increase in decay time especially in the mixed scenario or in the scenario in which all receptors are located inside of the nanocolumn. This may be explained by a greater frequency of recapture of glutamate molecules. It is clear from the results of Figure 4 that the impact of the crowding of the synaptic cleft by trans-synaptic filaments is highly dependant on the position of AMPA receptors as indicated by differences between scenarios in which all, half, or no receptors were located inside the nanocolumn. To further investigate this dependency, we designed an additional series of simulations. In this set of simulations, we placed the receptors on a circle which center coincides with the synapse center in such a way that the distance between the receptors and the synapse center is fixed while the receptors radial positions are chosen randomly (see Figure 5A). The number of receptors is set at 20, and their distance from synapse center ranges from 40 to 200 nm. The peak current (see Figures 5J–M) almost always reaches its maximum when all the receptors are placed close to the synapse center. However, for large numbers of released glutamate molecules (see Figure 5M), the peak current reaches its maximum when the distance between the receptor and the synapse center is about 50 nm. The effect of receptor location was weaker for larger numbers of released glutamate molecules.

We also paid attention to the proportion of neurotransmitter (glutamate) molecules that bind at least once (Figures 5N–Q) to an AMPA receptor. If this proportion is large, this can be viewed as an indication that the system is efficient and resources are not wasted. We found that this proportion decreases when receptors are placed further away from the center of the synapse. This is especially true when the anisotropy coefficient is equal to 0.9. In this case, glutamate molecules will have a very long dwelling time near the center of the synapse and will have little chance of being captured once they leave the synapse center. The rise time increases with the distance between the AMPA receptors and the synapse center especially when the anisotropy coefficient is very large (Figures 5B–E). Indeed, when the anisotropy coefficient is large, glutamate molecules take a longer time to leave the nanocolumn and to reach receptors located at the periphery. Finally, we investigated the impact of receptor location on the decay time of AMPA currents (Figures 5F–I). Decay time tends to be large when the distance between the synapse center and channels is small, especially when the anisotropy coefficient and nnt are large. This is due to the fact that crowding of the synaptic cleft by trans-synaptic molecules will force the glutamate molecules to remain longer at the center of the synapse. These molecules are then likely to bound several times to AMPA receptors located at the center of the synapse leading to a larger decay time. Results shown in Figures 5B–E,N–Q suggest that nanocolumns can play two opposite roles on synaptic signaling. On one hand, a nanocolumn can increase the probability that glutamate molecules are captured by the receptors located inside of the nanocolumn. This increase is due both to the decrease of the distance between the receptor release site and receptors and to the slowing of glutamate molecule movement within the nanocolumn. On the other hand, a nanocolumn can delay the capture of glutamate molecules by randomly distributed AMPA receptors possibly reducing the synaptic current.

### Impact of nanocolumn size on synaptic current

**Figure 6:**
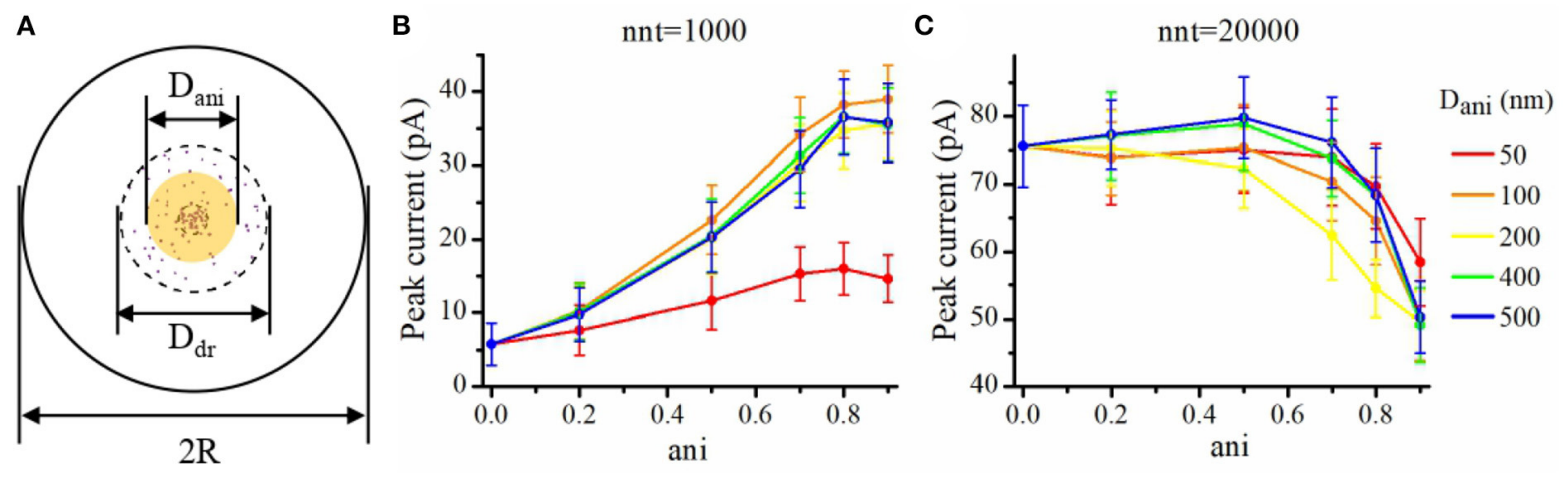
Influence of the size of the nanocolumn on peak current. **(A)** Schematic of the area where the diffusion of glutamate molecules is affected by trans-synaptic filaments (yellow shaded area). In this set of simulations, we set *D*_dr_ = 400 nm, *R* = 1, 000 nm, *R*_std_ = 50 nm, *n*_c_ = *n*_r_ = 40, and *H*_c_ = 20 nm. Peak synaptic current as a function of anisotropy coefficient (ani) when *D*_ani_ = 50 nm, *D*_ani_ = 100 nm, *D*_ani_ = 200 nm, *D*_ani_ = 400 nm, and *D*_*ani*_ = 500 nm for nnt = 1, 000 **(B)** and for nnt = 20, 000 **(C)**. Mean values were taken over 1,000 and 50 repetitions, respectively. The error bars represent the standard deviation.

**Figure 7:**
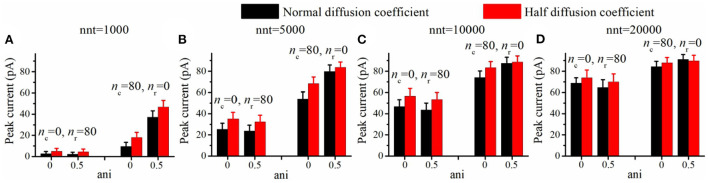
Increasing viscosity enhances peak current. Peak current as a function of the anisotropy coefficient (ani) for **(A)** 1,000, **(B)** 5,000, **(C)** 10,000, and **(D)** 20,000 released glutamate molecules. Black columns are obtained with normal neurotransmitter diffusion coefficient (0.30 μm^2^/ms) and red ones are obtained with reduced neurotransmitter diffusion coefficient (0.15 μm^2^/ms). The relative increase in peak current (red bar/black bar) is more important in the absence of a nanocolumn (ani = 0) than when a nanocolumn is including in the model (ani = 0.5). For example, the ratio of peak current for half diffusion coefficient to peak current for normal diffusion coefficient is 1.27 when ani = 0 but only 1.05 when ani = 0.5 (nnt = 5, 000, *n*_c_ = 80, *n*_r_ = 0). The error bars mark the standard deviation. The mean value is taken over 1, 000, 000/nnt repetitions. Other parameters were set to *R*_std_ = 50 nm, *D*_ani_ = 100 nm, and *H*_c_ = 20 nm.

We now investigate the impact of the size of the nanocolumn on synaptic currents. In our model, the size of the nanocolumn is parameterized by *D*_ani_ which stands for the diameter of the zone in which trans-synaptic proteins hinder longitudinal diffusion (see Figure 6A). In simulations described this far, we have fixed the diameter of the nanocolumn (*D*_ani_ = 100 nm). We now investigate how our results are modulated by this parameter. We monitor how the peak current varies as a function of the anisotropy coefficient for different values of *D*_ani_. All simulations were performed with other parameters fixed to *n*_c_ = *n*_r_ = 40 and *R*_*std*_ = 50 nm. For a small number of released glutamate molecules (when nnt = 1, 000) and for all values of *D*_ani_, the peak current increases as a function of the anisotropy coefficient (Figure 6B). Moreover, the peak current is largest when the size of the anisotropic diffusion zone (*D*_ani_) is 100 nm. Furthermore, when the *D*_ani_ is large, the influence of the size of nanocolumn becomes very small. The impact of the parameter *D*_ani_ = 50 nm is most noticeable when we compare the current obtained with the very small value of *D*_ani_ = 50 nm to the current obtained other scenarios. We also investigated the impact of the parameter *D*_ani_ when the number of released glutamate molecules was very large (nnt = 20, 000). In this case, we found that for all values of investigated *D*_ani_, the peak current decreases for very large values of anisotropy coefficients (Figure 6C). By showing that the results are modulated by the size of the zone in which trans-synaptic filaments hinder diffusion, we show that the effect of these filaments are distinct from a global decrease in diffusion coefficient (which could result from the addition of Dextran to the extracellular space for instance; see Figure 7).

### Impact of the synaptic electric field

Trans-synaptic currents create an electric field within the synapse which leads to the synapse center being slightly more depolarized compared to the rest of the extracellular space. The extent of this synaptic depolarization is dependent on current density and is expected to be larger when postsynaptic channels are densely concentrated as is the case when a nanocolumn is present. This prompted us to study the synaptic potential, its impact on synaptic current and how it is modulated by the presence of a nanocolumn. As discussed in the Method section, we model the electric field in the synaptic cleft assuming that the electric potential is equal to 0 mV at the outer boundary of the synaptic cleft and that the intracellular potential is constant at resting potential of −65 mV (Savtchenko et al., 2000). We choose to consider the intracellular potential as constant since accurately describing the postsynaptic response would require a detailed model of the postsynaptic neuron which we felt is beyond the scope of the present work. An example of how the electric potential varies with time is given in Supplementary Video in the form of a movie. We give two examples of the spatial distribution of the electric potential at maximal depolarization in Supplementary Figures 4A,B. We contrasted two scenarios, one in which all the AMPA receptors are outside of the nanocolumn (Supplementary Figure 4A) and one in which all the AMPA receptors are inside the nanocolumn (Supplementary Figure 4B). As expected, the depolarization occurring at the center of the synapse is larger when the AMPA receptors are concentrated inside the nanocolumn. Furthermore, in the case of *n*_c_ = 80 and *n*_r_ = 0, the maximal depolarization is near 5.49 mV which is large enough to have a significant impact on synaptic current.

**Figure 8:**
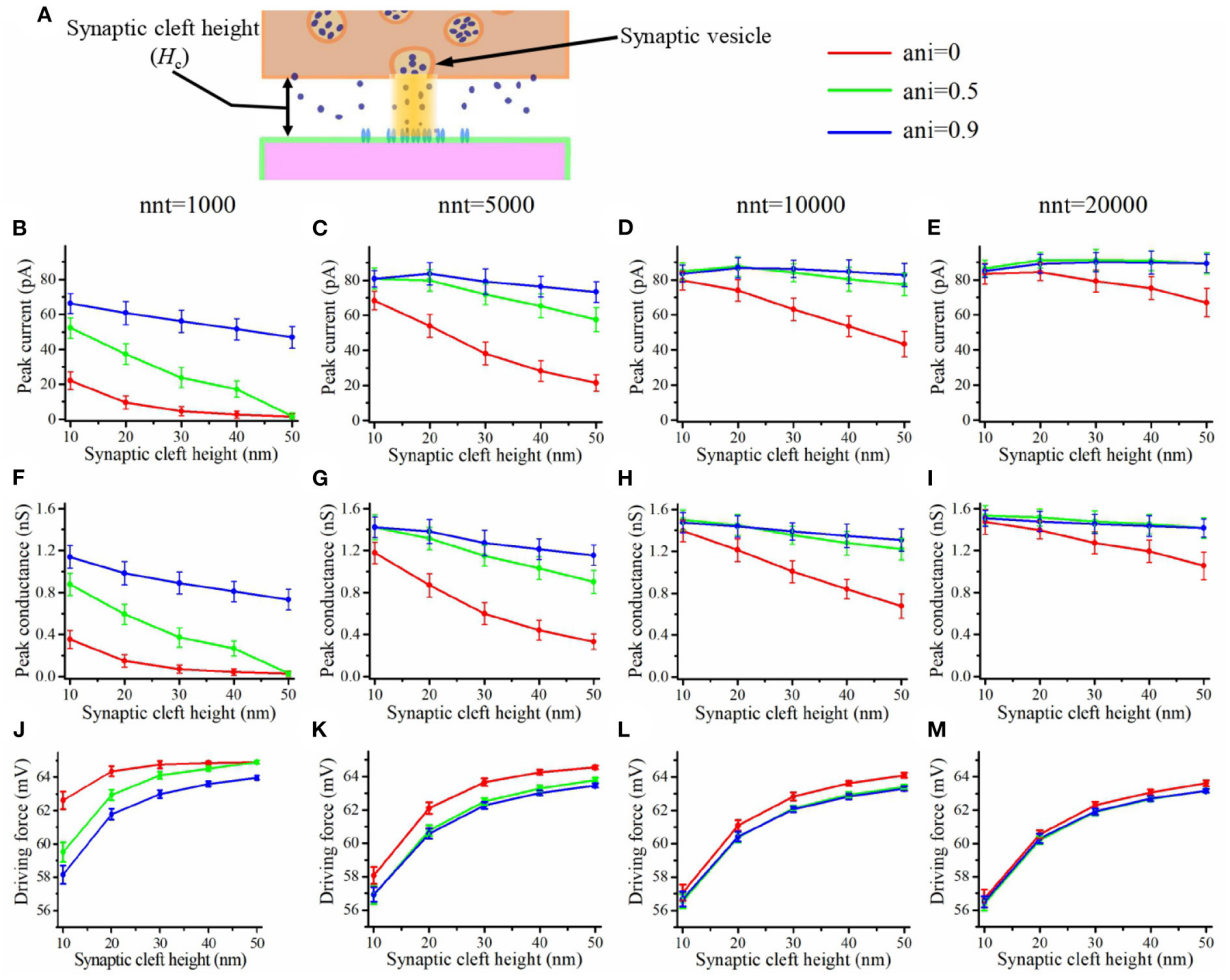
Influence of the synaptic cleft height. **(A)** Schematic illustrating the synaptic cleft height. Peak synaptic current as a function of synaptic cleft height for different number of released glutamate molecules [**(B)** nnt = 1, 000, **(C)** nnt = 5, 000, **(D)** nnt = 10, 000, and **(E)** nnt = 20, 000]. Peak synaptic conductance as a function of synaptic cleft height for different number of released glutamate molecules [**(F)** nnt = 1, 000, **(G)** nnt = 5, 000, **(H)** nnt = 10, 000, and **(I)** nnt = 20, 000]. Effective driving force (ratio of peak current to peak conductance) as a function of synaptic cleft height for different number of released glutamate molecules [**(J)** nnt = 1, 000, **(K)** nnt = 5, 000, **(L)** nnt = 10, 000, and **(M)** nnt = 20, 000]. The error bars mark the standard deviation. The mean value is taken over 1, 000, 000/nnt repetitions. For this set of simulations, we use 80 receptors, all located inside the nanocolumn, as well as the following parameter values *R*_std_ = 50 nm and *D*_ani_ = 100 nm.

The depolarization occurring at the center of the synaptic cleft leads to a decrease in the amplitude of trans-synaptic current by decreasing the driving force. This effect is especially important for small values of synaptic cleft height or for a high density of channels at the center of the synapse as may be caused by the presence of a nanocolumn. This depolarization within the synaptic cleft could lead to a decoupling between synaptic conductance and synaptic current. In Figure 8, we see that the peak conductance decreases monotonously as a function of synaptic cleft height (Figures 8F–I). On the other hand, the driving force increases with the cleft height as a large synaptic cleft leads to a smaller depolarization at the center of the synapse. This leads to the cleft height having a smaller impact on the peak current (Figures 8B–E) than on peak conductance. It has been argued that current would decrease for smaller values of cleft height due the large depolarization inside the cleft (Savtchenko and Rusakov, 2007). We didn't observe this since we didn't investigate cleft heights smaller than 10 nm.

Another potential effect of the electric field within the synapse occurs through the longitudinal electric gradient as it could impact the displacement of charged neurotransmitter molecules such as glutamate molecules. In order to investigate this effect, we compared the results of simulations accounting for the charge of glutamate and of simulations neglecting this charge. The relative impact of taking the charge of glutamate into account on either the synaptic current, the synaptic conductance or the concentration of glutamate molecules, is minimal as it remains below 1% in all cases investigated in the present paper.

### Impact of release site location and how this is modulated by the presence of a nanocolumn

**Figure 9:**
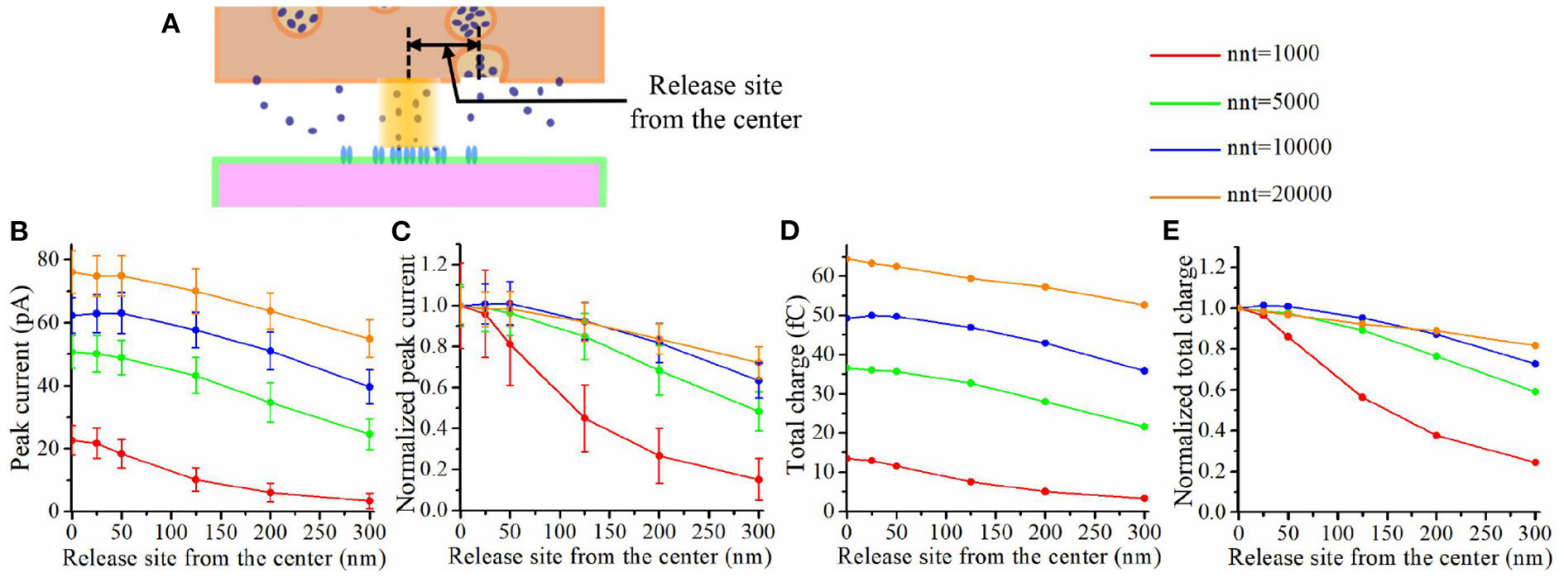
Impact of release site location. **(A)** Schematic illustrating the release site location. **(B)** Peak current as a function of the distance between the center of the synapse and the release site of the glutamate molecules. **(C)** Normalized peak current as a function of the distance between the center of the synapse and the release site of the glutamate molecules. **(D)** Total charge transfer as a function of the distance between the center of the synapse and the release site of the glutamate molecules. **(E)** Normalized total charge transfer as a function of the distance between the center of the synapse and the release site of the glutamate molecules. The error bars mark the standard deviation. The mean value is taken over 1, 000, 000/nnt repetitions. For this set of simulations, we use 40 receptors located inside the nanocolumn as well as 40 receptors distributed outside of the nanocolumn. We also used the following parameter values: *R*_std_ = 50 nm, ani = 0.5, *H*_c_ = 20 nm, and *D*_ani_ = 100 nm.

The impact of a nanocolumn on synaptic current is due to a large extent to the alignment of the receptors with the release site. We thus expect that this impact would be greatly affected by the location of the release site. To investigate this issue, we first varied the distance between the release site and the synapse center from 0 to 300 nm and paid attention to how this influences the peak current and the total charge transfer (see Figure 9A). Figure 9B shows that the peak current decreases with the distance between the release site and synapse center no matter the number of glutamate molecules released. Moreover, this effect was more important when a small number glutamate molecules were released (see Figure 9C). This suggests that the submicroscopic placement of the release site has a strong impact on the peak current for small vesicles. The trends we observe when looking at the total charge transfer are very similar to the effects on peak current (see Figures 9D,E).

**Figure 10:**
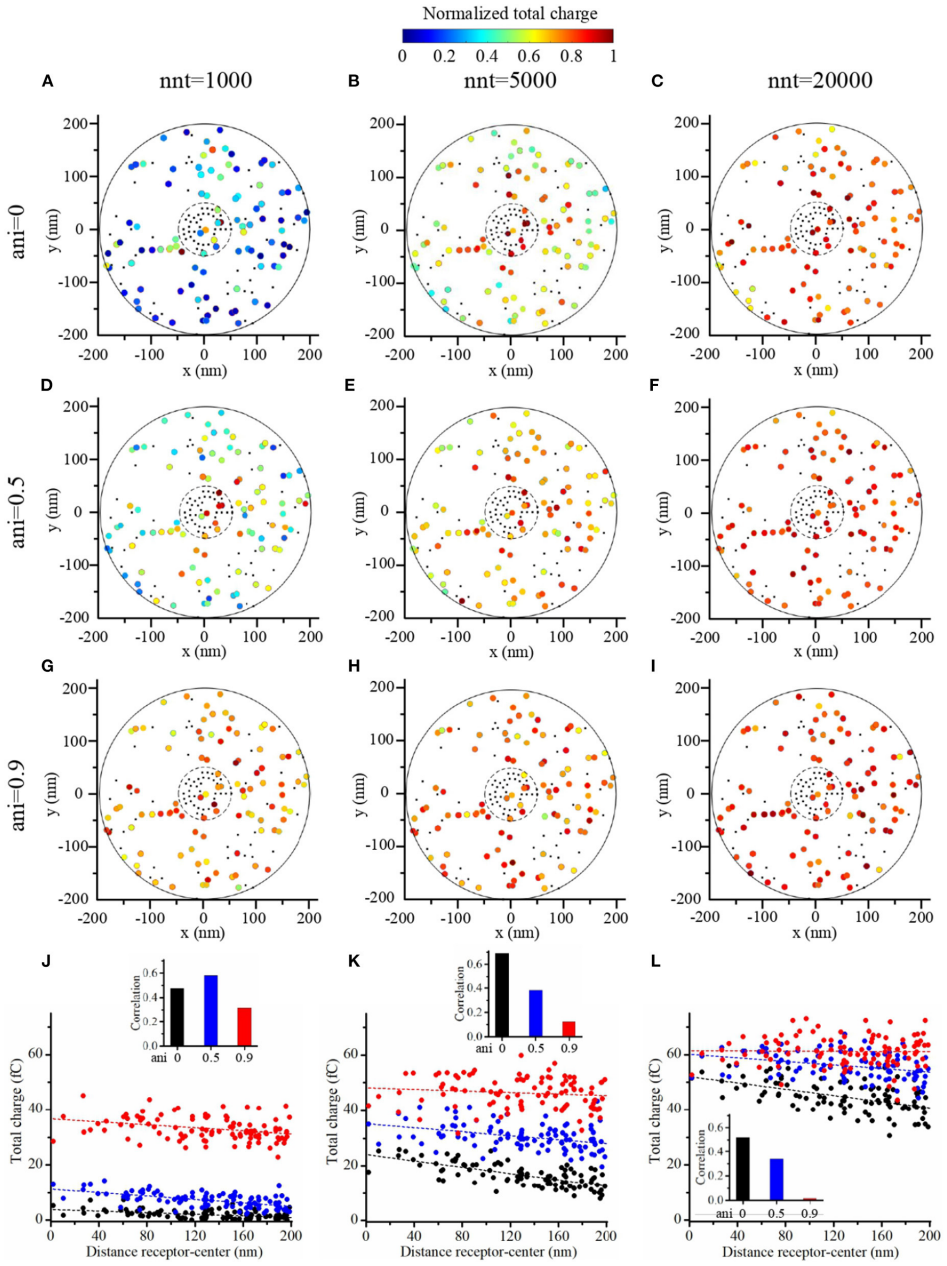
Random placement of vesicle openings. **(A–I)** We simulated the release of glutamate molecules at random locations within the postsynaptic dense area (PSD) to mimic the situation where the docking site would take place outside of the nanocolumn. The total charge transfer occurring during the first 2 ms after the release of glutamate molecules is color-coded. We contrasted a scenario in which we considered small vesicles (1,000 glutamate molecules) **(A,D,G)**, to a scenario in which we considered medium vesicles (5,000 glutamate molecules) **(B,E,H)** and one in which we considered large vesicles (20,000 glutamate molecules) **(C,F,I)**. Simulations were performed with three different values of anisotropy : 0 **(A–C)**, 0.5 **(D–F)**, and 0.9 **(G–I)** and 100 events are simulated in each set of simulations. The positions of receptors are the same in each set of simulations and are indicated by black dots. We use 40 receptors located inside of the nanocolumn and 40 receptors located outside of the nanocolumn. In each case, the total charge transfer is normalized to the maximal value in the set of simulations. **(J–L)** For illustrative purpose, we show the total charge transfers as a function of the distance between release sites and the synapse center. The points shown in **(J–L)** are taken from set **(A,D,G)**, set **(B,E,H)**, and set **(C,F,I)**, respectively. For each scenario, we computed the correlation between the distance from the synapse center to the release site and the total charge transfer. This correlation is always negative and we show its absolute value. In this figure, we use *R*_std_ = 50 nm, *D*_ani_ = 100 nm, and *H*_c_ = 20 nm.

To further investigate the impact of the release site location, we compared two scenarios. A first scenario in which the vesicle location is randomly distributed within the postsynaptic dense area, i.e., the docking site does not align with the nanocolumn. And a second scenario in which the release of glutamate molecules systemically occurs at the center of the synapse (i.e., the docking site is aligned with the nanocolumn). In first scenario, for different numbers of released glutamate molecules and anisotropy coefficient, we simulated 100 events in which the vesicle location was chosen randomly with a uniform distribution in the postsynaptic dense area (Figures 10A–I). In all cases, the total charge transfer was correlated with the distance between the release site and the synapse center (Figures 10J–L). The presence of a nanocolumn decreases the influence of the release site location on the total charge transfer, especially when the number of released glutamate molecules is large. Again, this points to the idea that for weak synapses (small presynaptic vesicle) the nano alignment of receptors and the anisotropy coefficient are important modulators of the synaptic responses. However, for large numbers of neurotransmitter molecules saturating the synapse, the nanometric location of receptors loses some of its significance.

### Changing the binding affinity modulates the impact of nanocolumn

Having investigated the impact of the various parameters characterizing the nanocolumn and the release site, we now turn to study if the presence of a nanocolumn could modulate the synapse response to drugs targeting AMPA channels. AMPA receptors are involved in many pathologies such as amyotrophic lateral sclerosis, alzheimer's, epilepsy and ischemia (Chang et al., 2012). Many drugs acting on synaptic transmission do so by changing the affinity of receptors, that is by making them more or less likely to bind with neurotransmitter molecules. A question of interest is how the presence of a nanocolumn would modulate the effect of drugs affecting the affinity of AMPA receptors. This preliminary investigation could reveal if nanocolumns are likely to make pharmacological treatments more or less effective. To model the effect of drugs affecting the affinity of AMPA receptors to glutamate neurotransmitter molecules, we changed proportionally the two rate constants *K*_1_ and *K*_2_. These two constants describe the main binding affinities and explicitly specify the transition rates from state C0 to C1 and from state C1 to C2, respectively. In a first set of simulations, we replaced these rate constants by half their normal values mimicking the impact of a drug partially preventing glutamate binding. In another set of simulations, we replaced these constants by twice their normal values mimicking the impact of a drug promoting synaptic transmission by favoring the binding of glutamate molecules to AMPA receptors.

**Figure 11:**
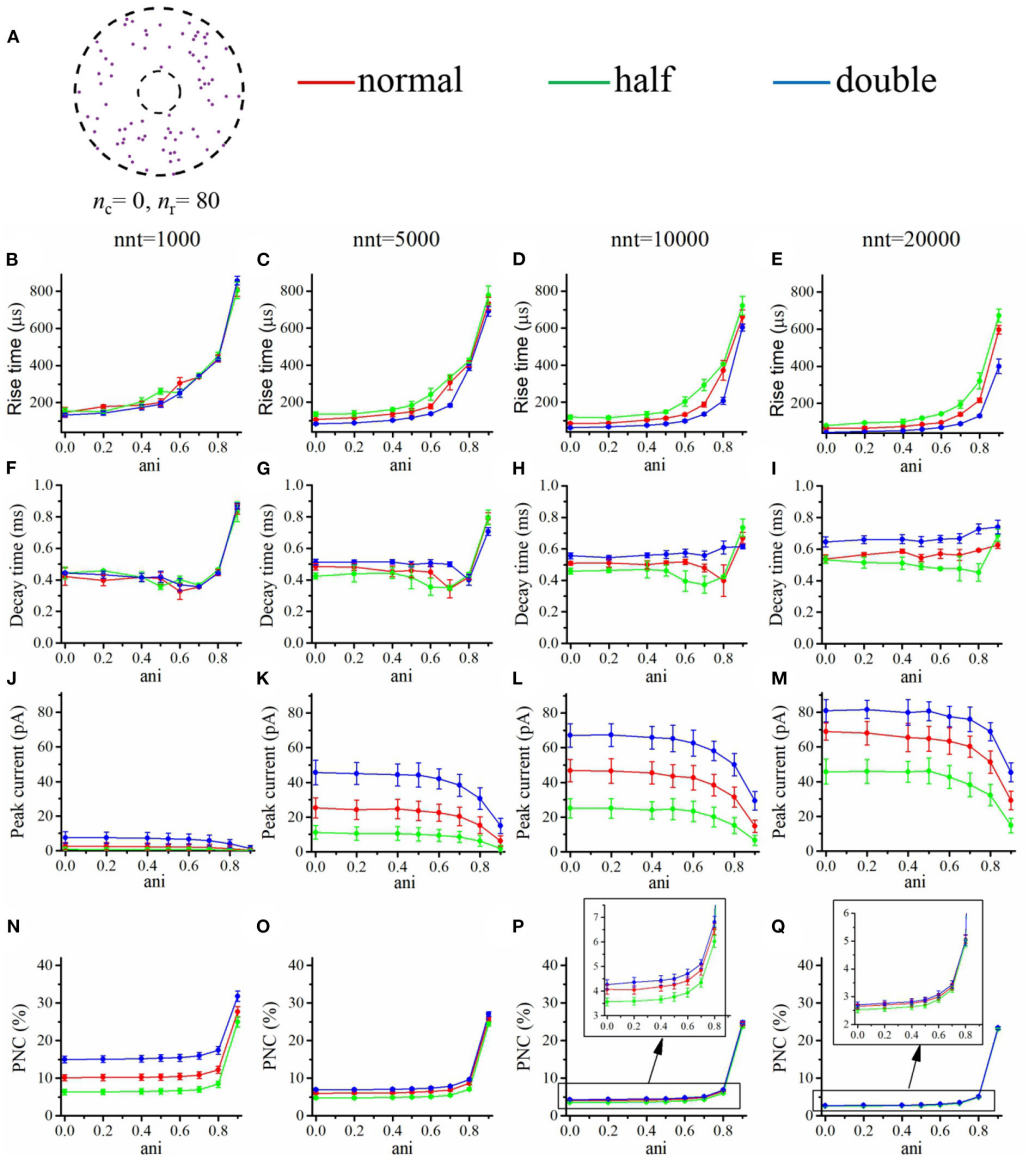
Changing the binding affinity in a scenario where all AMPA receptors are located outside the nanocolumn. **(A)** Receptor distribution for *n*_c_ = 0 and *n*_r_ = 80. Rise time as a function of anisotropy coefficient (ani) for different numbers of released glutamate molecules [**(B)** nnt = 1, 000, **(C)** nnt = 5, 000, **(D)** nnt = 10, 000, and **(E)** nnt = 20, 000]. Decay time as a function of anisotropy coefficient (ani) for different numbers of released glutamate molecules [**(F)** nnt = 1, 000, **(G)** nnt = 5, 000, **(H)** nnt = 10, 000, and **(I)** nnt = 20, 000]. Peak current as a function of anisotropy coefficient (ani) for different number of released glutamate molecules [**(J)** nnt = 1, 000, **(K)** nnt = 5, 000, **(L)** nnt = 10, 000, and **(M)** nnt = 20, 000]. Proportion of neurotransmitter molecules captured (PNC) as a function of the anisotropy coefficient (ani) for different number of released glutamate molecules [**(N)** nnt = 1, 000, **(O)** nnt = 5, 000, **(P)** nnt = 10, 000, and **(Q)** nnt = 20, 000]. The red curves are obtained with normal binding rates, the green curves with half binding rates, and the blue ones with double binding rates. The error bars mark the standard deviation. The mean value is taken over 1, 000, 000/nnt repetitions. For this set of simulations, we set *R*_std_ = 50 nm, *D*_ani_ = 100 nm, and *H*_c_ = 20 nm.

**Figure 12:**
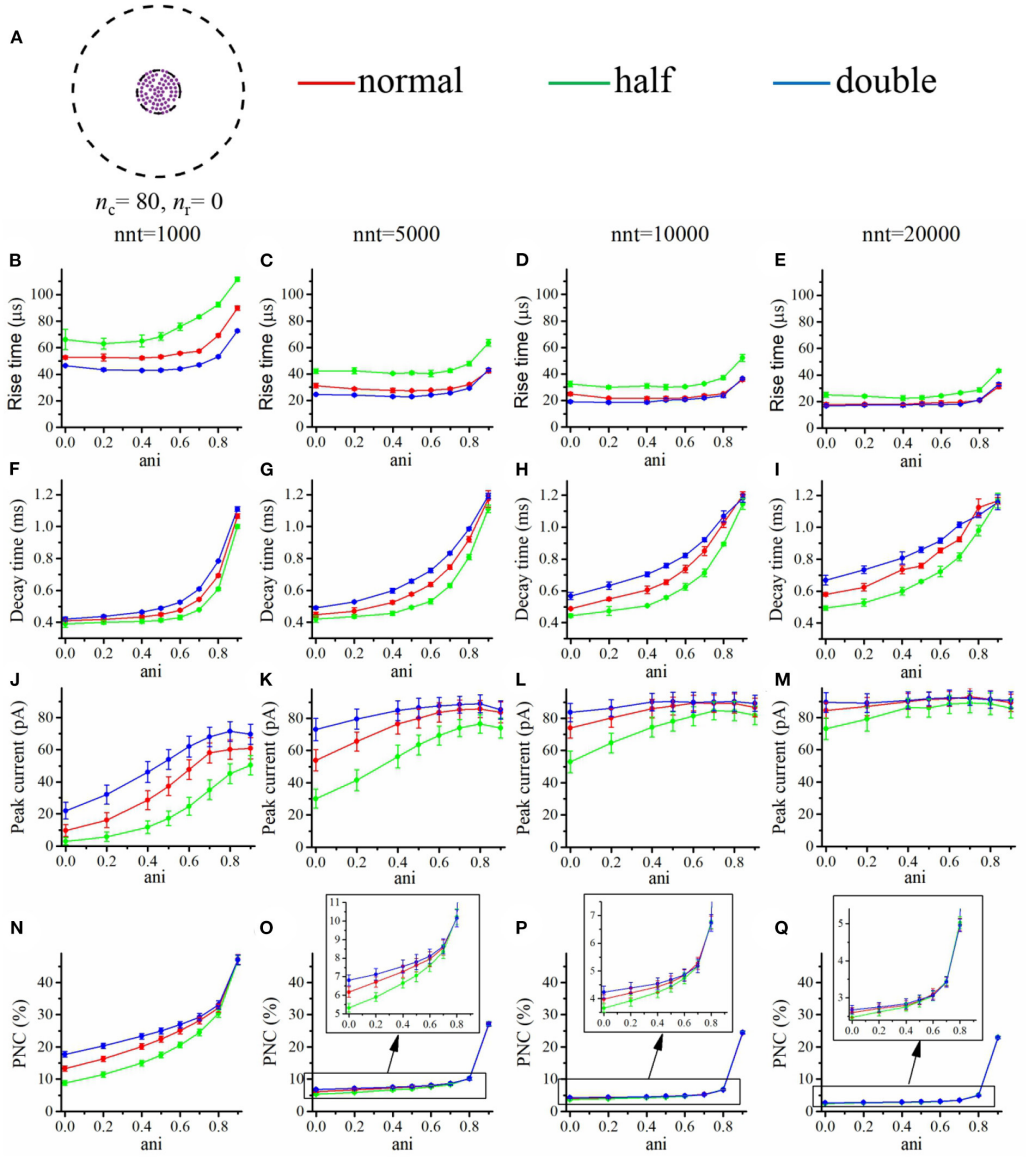
Changing the binding affinity in a scenario where all receptors located inside the nanocolumn. **(A)** Receptor distribution for *n*_c_ = 80 and *n*_r_ = 0. Rise time as a function of anisotropy coefficient (ani) for different numbers of released glutamate molecules [**(B)** nnt = 1, 000, **(C)** nnt = 5, 000, **(D)** nnt = 10, 000, and **(E)** nnt = 20, 000]. Decay time as a function of anisotropy coefficient (ani) for different numbers of released glutamate molecules [**(F)** nnt = 1, 000, **(G)** nnt = 5, 000, **(H)** nnt = 10, 000, **(I)** nnt = 20, 000]. Peak current as a function of anisotropy coefficient (ani) for different number released glutamate molecules [**(J)** nnt = 1, 000, **(K)** nnt = 5, 000, **(L)** nnt = 10, 000, and **(M)** nnt = 20, 000]. Proportion of neurotransmitter molecules captured (PNC) as a function of the anisotropy coefficient (ani) for different number of released glutamate molecules [**(N)** nnt = 1, 000, **(O)** nnt = 5, 000, **(P)** nnt = 10, 000, and **(Q)** nnt = 20, 000]. The red curves are obtained with normal binding rates, the green curves with half binding rates, and the blue curves with double binding rates. The error bars mark the standard deviation. The mean value is taken over 1, 000, 000/nnt repetitions. For this set of simulations, we use *R*_std_ = 50 nm, *D*_ani_ = 100 nm, and *H*_c_ = 20 nm.

Our goal was to investigate how the impact of these changes in affinity were modulated by the presence of a nanocolumn. We thus tested two contrasted scenarios, in the first one (Figure 11), all channels were distributed as in the absence of a nanocolumn, while in the second scenario (Figure 12), all channels are located inside the nanocolumn. As expected, in all cases, increasing the affinity increased the peak current. Of particular interest is the scenario in which all the receptors are within the nanocolumn and the number of released glutamate molecules is small (nnt = 1, 000; Figure 12J). We see that the relative change in peak current due to changing the affinity is smaller when the anisotropy is high, indicating that a strong nanocolumn may mitigate the effect of drugs changing the affinity.

### Lateral diffusion of AMPA receptors

**Figure 13:**
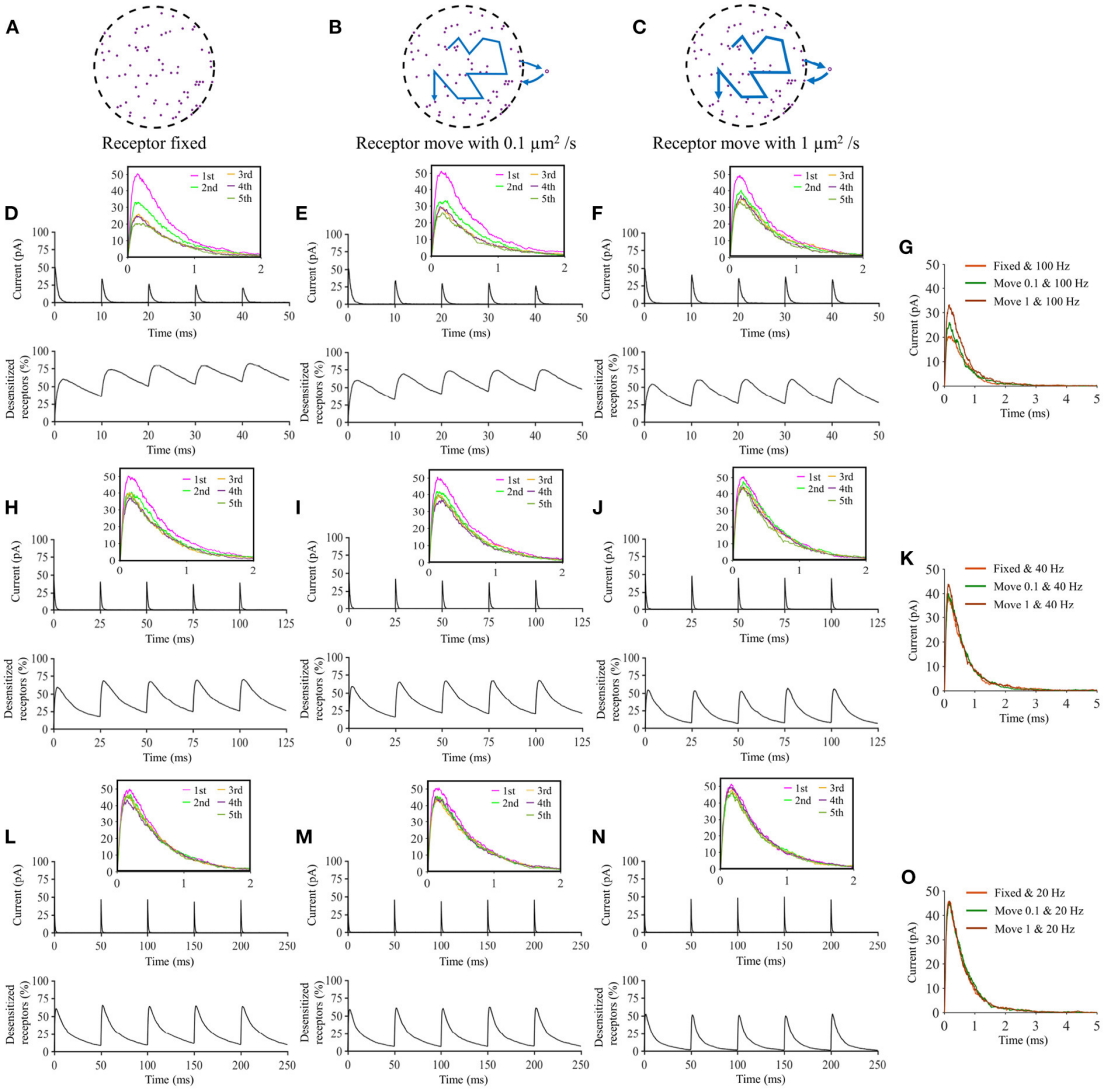
Impact of lateral diffusion of AMPA receptors without a nanocolumn. The schematic of 80 receptors randomly distributed in the PSD with different situation: **(A)** AMPA receptors fixed, **(B)** diffusion coefficient = 0.1 μm^2^/s, and **(C)** diffusion coefficient = 1 μm^2^/s. The top curves show the current as a function of time and the bottom ones show the percentage of desensitized receptors as a function of time for different release frequencies [**(D–F)** Frequency = 100 Hz; **(H–J)** Frequency = 40 Hz, and **(L–N)** Frequency = 20 Hz]. We can compare the 5th event of **(D)** and **(F)** in **(G)** of **(H)** and **(J)** in **(K)** as well as of **(L)** and **(N)** in **(O)**. Mean values were taken over 20 repetitions. In this set of simulations, we set *R*_std_ = 50 nm, *D*_ani_ = 100 nm, ani = 0, nnt = 10, 000, and *H*_c_ = 20 nm.

Lateral diffusion of postsynaptic receptors is known to play an important role in synaptic potentiation and in synaptic signaling in general (Heine et al., 2008). It is been argued that lateral diffusion of postsynaptic receptors could prevent receptor desensitization in the event of repeated stimulation by effectively replenishing the pool of receptor under the vesicle (Choquet and Hosy, 2020). By adding stochastic diffusion of AMPA receptors to our model, we could test whether our model is able to replicate this effect. We first performed a set of simulations in which the postsynaptic receptors diffused freely. We simulated repeated high frequency presynaptic released and quantified the postsynaptic current response. We compared the results to a “control” scenario in which the postsynaptic receptors were fixed (see Figure 13). We observe that, as previously hypothesized (Choquet and Hosy, 2020) the lateral diffusion of receptors mitigated receptor diffusion which lead to higher current responses to a train of events.

**Figure 14:**
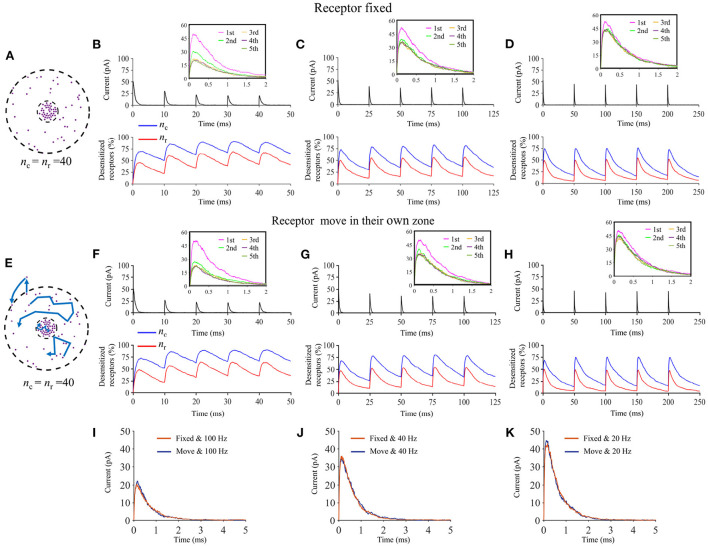
Impact of lateral diffusion of AMPA in the presence of a nanocolumn. **(A)** A schematic of the distribution of receptors for **(B–D)**. For this set of simulations **(B–D)**, we fixed the position of receptors during each repetition. The top curves show the current as a function of time and the bottom ones show the percentage of desensitized receptors as a function of time for different release frequencies. [**(B)** Frequency = 100 Hz, **(C)** Frequency = 40 Hz, and **(D)** Frequency = 20 Hz]. **(E)** Schematic distribution of AMPA receptors for **(F–H)**. For this set of simulations, AMPA receptors inside the nanocolumn move with diffusion coefficient of 0.01 μm^2^/s while the AMPA receptors outside the nanocolumn move with diffusion coefficient of 0.1 μm^2^/. The top curves show the current as a function of time and the bottom ones show the percentage of desensitized receptors as a function of time for different release frequencies. [**(B)** Frequency = 100 Hz, **(C)** Frequency = 40 Hz, and **(D)** Frequency = 20 Hz]. We can compare the 5th event of **(B)** and **(F)** in **(I)**, of **(C)** and **(G)** in **(J)** as well as of **(D)** and **(H)** in **(K)**. For this set of simulations,We use 40 receptors located inside of the nanocolumn and 40 receptors located outside of the nanocolumn. Mean values were taken over 20 repetitions. The distribution of receptor of the 20 repetitions may be different. We set *R*_std_ = 50 nm, *D*_ani_ = 100 nm, ani = 0.5, nnt = 5, 000, and *H*_c_ = 20 nm.

We found however, that the ability of postsynaptic receptor diffusion to prevent desensitization is highly dependant on the frequency of vesicle openings being significant only at very high frequencies (>40 Hz). This is related to the fact that in the absence of receptor diffusion, the receptors take about 25 ms on average to leave the desensitized state (see Markov chain description of channel transition Figure 1C and in particular the values of *K*_−7_, *K*_−9_, and *K*_−10_). For instance, in Figures 13H, 14C, we see that in the absence of receptor diffusion, the proportion of desensitized channels roughly decreases by two during 25 ms. For an effect of lateral diffusion to be observable, both the interval between releases and the time needed for a receptor to move out of the docking site vicinity must be smaller or of the order of ~25 ms. Figures 13, 14 suggest that, except for high frequency simulations (>40 Hz), desensitization has little impact on our simulation results. This justifies a posterior the focus on single vesicle openings throughout the paper.

Furthermore, we wanted to assess how the impact of AMPA receptor lateral diffusion is modulated by the presence of a nanocolumn. To do so, we performed an additional set of simulations in which we described the lateral diffusion of AMPA receptors in the presence of a nanocolumn. In these simulations, we assumed that the diffusion of receptors within the nanocolumn would be reduced. In order to facilitate the interpretation of the results, we wanted to maintain the proportion of receptors inside and outside the nanocolumn constant throughout the simulations. To achieve this, we imposed that the receptors within the nanocolumn would diffuse but stay within the nanocolumn while the receptors outside the nanocolumn would also diffuse but remain outside of the nanocolumn. In this scenario, whether the receptors were allowed to diffused or not made no significant difference on the synaptic current strength as shown in Figure 14.

## 4. Discussion

To study *in silico* the functional influence of nanocolumns on synaptic currents, we simulated a glutamatergic synapse with AMPA receptors. We investigated how features of synaptic currents such as amplitude, rise time, and decay time depend on parameters related to nanocolumn properties such as the number of receptors in the nanocolumn and the hindrance on diffusion by trans-synaptic molecules (characterized by the anisotropy coefficient). A natural hypothesis is that the presence of a nanocolumn would lead to an increase in the amplitude of trans-synaptic current by decreasing the distance between receptors and the site of glutamate molecule release. We also believed *a priori* that this greater proximity between the glutamate molecule release site and the AMPA receptors could decrease the rise time of synaptic events. We found that while these hypotheses are partly true, the full picture is somewhat more complex.

We numerically demonstrated that the presence of a nanocolumn could indeed enhance peak synaptic current though the extent of this effect is highly dependent on both the number of release glutamate molecules and the number of AMPA receptors (Figure 4). In some scenarios, when the trans-synaptic filaments greatly hinder the diffusion of glutamate molecules and when the receptors are not tightly distributed, the synaptic current was shown to be diminished by the presence of a nanocolumn. Moreover, the impact of a nanocolumn on synaptic current is limited when the number of the number of released glutamate molecules is large. On the other hand, when the number of AMPA receptors is large and when the number of released glutamate molecules is relatively small, the presence of a nanocolumn significantly increases synaptic peak current. It does so by increasing the concentration of glutamate molecules in the vicinity of the AMPA receptors. This indicates that the existence of nanocolumns can help the system reduce the number of neurotransmitter molecules necessary to trigger a postsynaptic response which could decrease the energy consumption of the presynaptic neuron. This also suggests that by reinforcing the currents in weak synapses, nanocolumns could possibly play a role in their potentiation.

With our *in silico* model, we also investigated other aspects of synaptic transmission such as the numbers of transitions between individual channel states. According to our model of AMPA receptors, one receptor can open several times, and the most frequent state transitions occur between the open state “O2” and the closed bound state “C2.” Recapture of neurotransmitter molecules by channels after unbinding is also described in our model. We show that for small vesicles, since up to 16% of glutamate molecules can be captured at least once (Figure 3), the binding of glutamate molecules to AMPA channels can have an significant impact on the concentration of free (available) glutamate molecules. We also computed the electric field occurring within the synaptic cleft together with its impact on glutamate diffusion and trans-synaptic current (Supplementary Figure 4). According to the previous works, very small values of synaptic cleft height promote conductance by slowing diffusion of neurotransmitter molecules but can also be detrimental to synaptic current by creating a strong electric field within the synaptic cleft (Savtchenko and Rusakov, 2007) which decreases the driving force. We found that the extent of the electrical depolarization within the cleft and its detrimental effect on the synaptic driving force is increased by the presence of a nanocolumn.

We also performed simulations to investigate the impact of lateral diffusion of postsynaptic receptors on synaptic currents. It was previously proposed (Choquet and Hosy, 2020) that the lateral diffusion of postsynaptic receptors could mitigate desensitization of receptors and thus partially prevents the decline of current amplitudes during a train of synaptic events. While we could replicate this effect in our simulations, we could only observe it for very high frequencies of presynaptic release frequencies. When assuming the presence of a nanocolumn which would slow the diffusion of receptors within it, then the impact of receptor lateral diffusion on preventing desensitization became non-significant. The quantitative results obtained here are dependant on the type of receptors (AMPA) and neorotransmitter (glutamate) described in the model. It would be of interest in future works to investigate the impact of post-synaptic receptors lateral diffusion in models including other types of receptors such as NMDA for instance.

On the methodological side, our work suggests three important points. (1) There is an advantage to describe the individual movements, bindings and unbindings of single neurotransmitter molecule. (2) There is also a decoupling between the conductance and the current due to the existence of an electric depolarization within the synaptic cleft. Thus, the synaptic electric field needs to be included in the model. (3) The effect of the electric field on the movement of electrically charged neurotransmitter molecules does not have a significant impact on the resulting trans-synaptic current.

Although we focused our attention on a glutamatergic synapse with AMPA receptors, the formalism developed in the present manuscript could be applied to a wide variety of synapses. There are also several extensions that could be added to the present model. First, one could try to model the effect of the presence of several nanocolumns in the same synapse. It is not *a priori* obvious what type of results this investigation would yield as the neurotransmitter molecules could spill from one nanocolumn to another making the end result difficult to predict and to interpret. As our results indicate that nanocolumns could play a role in promoting synaptic current in small synapses, these structures could be involved in synaptic potentiation. It would thus be interesting to incorporate nanocolumns in more complete models of dendritic spines that would be able to describe short term and long term synaptic potentiation. Model extensions could also describe the formation of nanocolumns. We hope that this work will encourage more investigation in understanding the functional role of nanocolumns and that the novel elements developed in our manuscript will be useful to future research.

## Data availability statement

The raw data supporting the conclusions of this article will be made available by the authors, without undue reservation.

## Author contributions

XL developed the model and its Matlab implementation, performed the simulations and generated the figure, and wrote and revised the paper. GH developed the model and its Matlab implementation. AGG contributed to the design of the research, performed simulations, and wrote and revised the paper. ND developed the model and wrote and revised the paper. All authors contributed to the article and approved the submitted version.

## Funding

AGG is a Scholar of the Fonds de recherche du Québec—Santé and was supported by a Sentinel North Partnership Research Chair. We also acknowledge support from the Natural Sciences and Engineering Research Council of Canada (AGG #06507 and ND #04483).

## Conflict of interest

The authors declare that the research was conducted in the absence of any commercial or financial relationships that could be construed as a potential conflict of interest.

## Publisher's note

All claims expressed in this article are solely those of the authors and do not necessarily represent those of their affiliated organizations, or those of the publisher, the editors and the reviewers. Any product that may be evaluated in this article, or claim that may be made by its manufacturer, is not guaranteed or endorsed by the publisher.
