# Supplementary material for: Computational modeling of trans-synaptic nanocolumns, a modulator of synaptic transmission
Source: Front Comput Neurosci. 2022 Sep 28;16:969119. doi: 10.3389/fncom.2022.969119 (PMC9554614; doi:10.3389/fncom.2022.969119)
Supplement: Supplementary file 1 [file Data_Sheet_1.pdf]

Supplementary Material

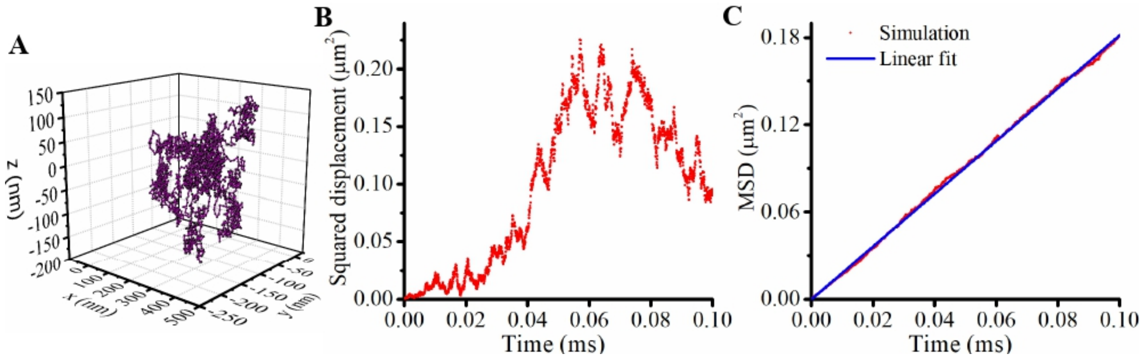

Figure S1. Simulations describing the diffusion coefficient. A Typical trajectory of a single glutamate molecule in an infinite 3D space within 0.1 ms. **B** Time course of squared displacement. Time course of MSD (mean squared displacement) (red curve), and it is obtained with a mean MSD of 400 trajectory. The blue line is a linear fitted curve of the red curve. According to the Einstein relation in 3D, $D = \lim_{t \to \infty} \frac{\mathrm{MSD}}{6t}$, we get the diffusion coefficient $D = 0.30\ \mu\mathrm{m}^2/\mathrm{ms}$.

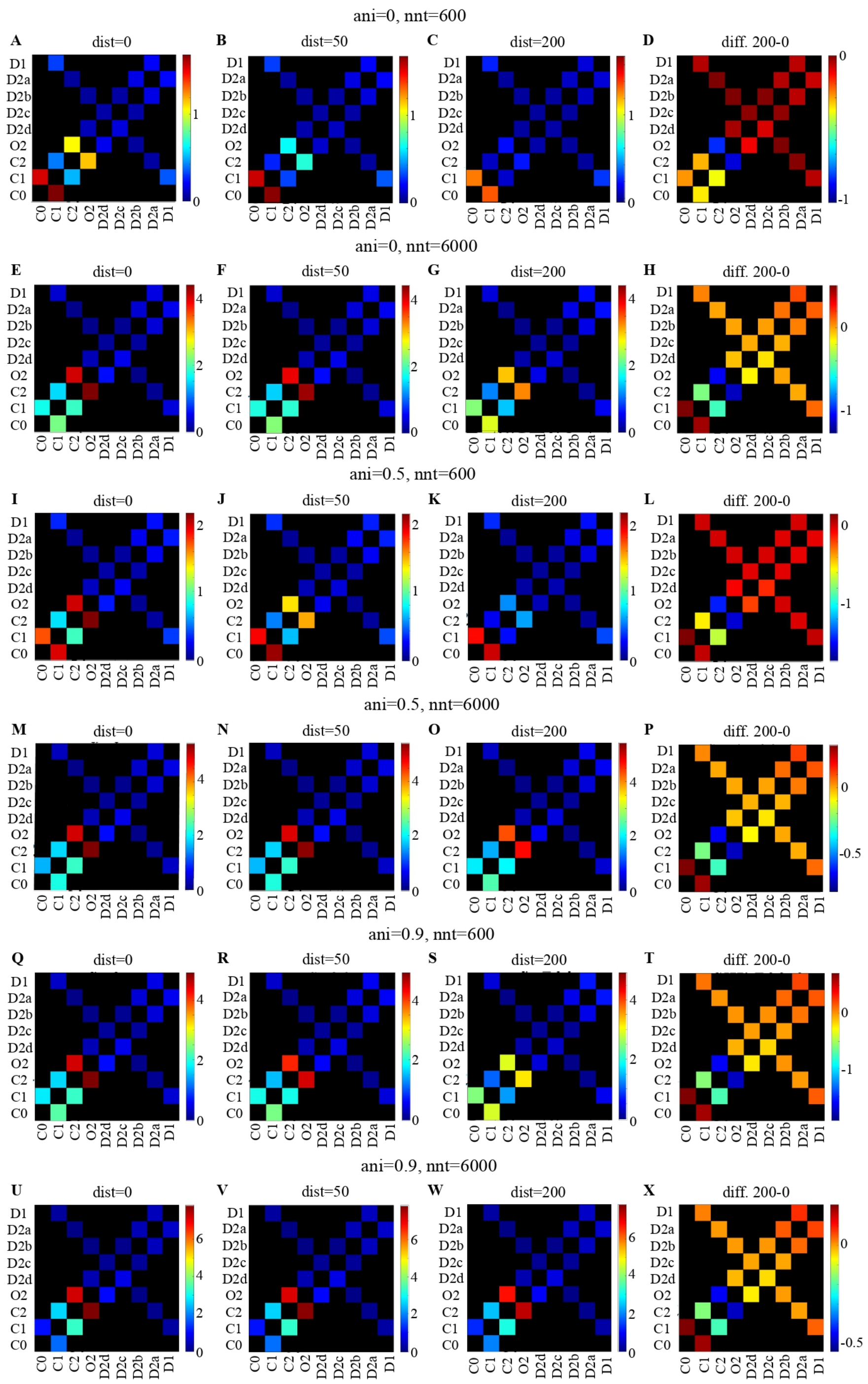

Figure S2. Deterministic model describing the frequency of receptor state transition. We use a deterministic model to compute the expected number of transitions between any two given channel states. We tested three channel positions, one with the channel located directly at the center of the synapse (left column) on with a channel located at 50 nm from the synapse center (second column from left) and one with a channel located at 200 nm from the synapse center (third column from left). We also tested two different number of glutamate neurotransmitter molecules (600 and 6000) as well as two values of anisotropy coefficient (0.5 and 0.9). The rightmost column displays differences between the scenario in which the channel is located at the synapse center and the scenario in which the channel is located at 200 nm from the synapse center. Observe that the most frequent transitions are between the open state (O2) and the close and bound state (C2).

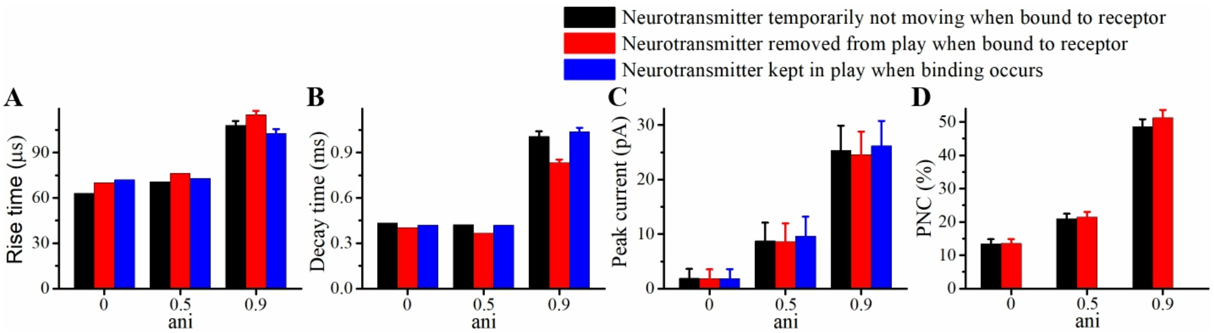

Figure S3. Impact of the glutamate neurotransmitter molecule capture modeling formalism. We simulated a synaptic response under three modeling formalisms used to describe the binding of neurotransmitters: 1. the neurotransmitter molecule is temporarily removed from the simulation for the time it is captured by a receptor (black), 2. the neurotransmitter molecule is removed from the simulation when it is captured (red) and 3. the neurotransmitter molecule stays in play while being captures (blue), which is analogous to the case where the concentration is kept constant during the simulation. **A** Rise time as a function of the anisotropy coefficient (ani). **B** Decay time as a function of ani. **C** Peak current as a function of ani. **D** Proportion of neurotransmitter (glutamate) molecules captured (PNC) as a function of ani. The error bars correspond the standard deviation. The mean value is taken over 500 repetitions. Other parameters were set to $n_\mathrm{c} = n_\mathrm{r} = 40$, $\mathrm{nnt} = 500$, $R_\mathrm{std} = 50$ nm, $D_\mathrm{ani} = 100$ nm and $H_\mathrm{c} = 20$ nm. We observe that the choice of modeling formalism impacts the rise time and decay time.

Supplemental video. We provide two movies showing the temporal evolution of the electric field in the synapse. For each of these movies, we used the following conditions A. 0 receptors inside the nanocolumn and 80 outside the nanocolumn B. 80 inside the nanocolumn and 0 outside the nanocolumn. Other parameters were set to $\mathrm{nnt} = 20000$, $R_\mathrm{std} = 50$ nm, $D_\mathrm{ani} = 100$ nm, $\mathrm{ani} = 0.5$ and $H_\mathrm{c} = 20$ nm .

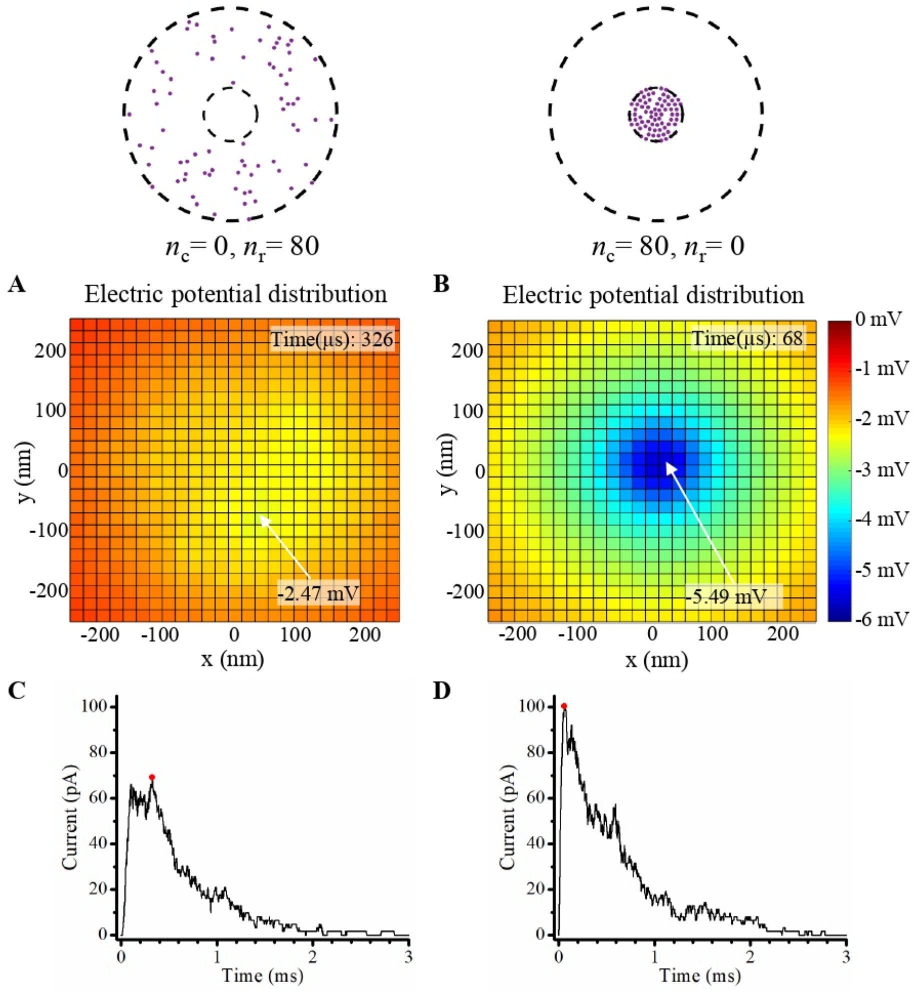

Figure S4. The synaptic cleft undergoes a depolarization during a synaptic event. Two examples of electric potentials at peak depolarization **A** and **B**. For **A**, we use 0 receptors located inside of the nanocolumn and 80 receptors located outside of the nanocolumn. For **B**, we use 80 receptors all located inside the nanocolumn as illustrated in the top insets. In panels **C** and **D**, we show the corresponding synaptic current as a function of time. The red dots indicate the time of the peak depolarization within the synapse. For **A** and **B** this is equal respectively to 326 μs and 68 μs, and the biggest voltage is -2.47 mV and -5.49 mV. Other parameters were set to $\mathrm{nnt} = 20000$, $R_{\mathrm{std}} = 50$ nm, $D_{\mathrm{ani}} = 100$ nm, $\mathrm{ani} = 0.5$ and $H_{\mathrm{c}} = 20$ nm.
